# Sumud as a framework for collective resilience: a systematic review of implications for transcultural psychiatry and psychotherapy

**DOI:** 10.3389/fpsyt.2026.1908428

**Published:** 2026-10-16

**Authors:** Dina Aly El-Gabry, Iffat Elbarazi, Emmanuel Stip, Reem Al Sheryani, Leena Amiri, Tarek Okasha, Nadia Taysir Dabbagh

**Affiliations:** 1 Department of Psychiatry, College of Medicine and Health Sciences, United Arab Emirates University, Al-Ain, United Arab Emirates; 2 Okasha Institute of Psychiatry, Neuropsychiatry Department, Faculty of Medicine, Ain Shams University, Cairo, Egypt; 3 Institute of Public Health, College of Medicine and Health Sciences, United Arab Emirates University, Al Ain, United Arab Emirates; 4 Health Education Research Unit, Institute of Public Health, College of Medicine and Health Sciences, United Arab Emirates University, Al Ain, United Arab Emirates; 5 Department of Psychiatry and Addictology, Faculty of Medicine, Université de Montréal, Montréal, QC, Canada; 6 Institut Universitaire en Santé Mentale de Montréal, Université de Montréal, Montréal, QC, Canada; 7 Public Services & Outreach Unit, United Arab Emirates University, Al Ain, United Arab Emirates; 8 Al Jalila Children’s Hospital, Dubai Health, Dubai, United Arab Emirates

**Keywords:** collective resilience, cultural psychiatry, global mental health, psychotherapy, resilience, sumud, transcultural psychiatry

## Abstract

**Background:**

Resilience is increasingly understood as a dynamic, ecological process; however, prevailing psychiatric models continue to emphasize individual traits, thereby neglecting collective and culturally embedded forms of endurance. Sumud (Arabic: صمود), broadly translating as steadfastness, perseverance, and endurance, sustains identity, dignity, and daily life under chronic adversity across the Arab world and functions as a relational and ethical orientation. This review examines sumud as a resilience framework for transcultural psychiatry and psychotherapy.

**Aims:**

To synthesize conceptualizations of sumud in relation to resilience, coping, and adaptation, and to evaluate implications for narrative, family-based, group, community-oriented, and arts-informed therapeutic approaches.

**Methods:**

A PRISMA 2020-guided systematic narrative review was conducted. Four electronic databases (PubMed, Scopus, Web of Science, and Embase) were searched covering publications up to 1 June 2026. English-language studies were eligible if they explicitly examined sumud as a cultural, psychological, or collective construct with relevance to resilience, coping, or psychotherapy. Eligible publication types included qualitative, quantitative, and mixed-methods empirical studies; theoretical and conceptual papers; ethnographies; narrative analyses; and reviews. Data were thematically synthesized using a predefined extraction matrix. The review was registered with PROSPERO (CRD420261279052).

**Results:**

Thirty-nine studies met the inclusion criteria. Descriptively sumud emerged as a multilevel, collective form of resilience enacted through family systems, intergenerational narratives, cultural practices, and institutional structures. Substantively, it supported meaning-making, emotional regulation, and identity continuity. Critically, ethical tensions and cumulative burdens were documented, particularly for women, caregivers, and professionals., and rigorously evaluated, sumud-informed psychotherapeutic interventions remain scarce, notwithstanding a general preference in mental health studies for social-ecological models and narrative, family-based, group, and arts-based therapeutic approaches.

**Conclusions:**

Theoretically, sumud challenges individualistic psychiatric frameworks by offering a culturally embedded, collective model of resilience. Clinically, it urges clinicians to engage with patients’ narratives, relational worlds, and cultural practices, while remaining attentive to the costs of endurance. Prospectively, in the context of ongoing conflicts and humanitarian crises affecting parts of the Middle East and Gulf region, sumud warrants renewed scholarly and clinical attention for the development of culturally responsive, resilience-informed psychotherapies.

**Systematic review registration:**

https://www.crd.york.ac.uk/prospero/, identifier CRD420261279052.

## 1 Introduction

Resilience is widely regarded as the capacity to adapt positively in the face of adversity, yet the concept remains heterogeneous and multifaceted. Foundational studies framed resilience primarily as an individual trait, highlighting personal strengths, coping mechanisms, and emotional regulation (1, 2). Subsequent research has shifted toward understanding resilience as a dynamic process shaped by the interplay of personal, relational, and environmental factors (3, 4). Denckla et al. argue that resilience must be approached as a multidimensional phenomenon unfolding across biological, psychological, and social systems, requiring integrative models that reflect this complexity (5). This evolution has supported broader ecological frameworks that attend to the roles of family, community, culture, and social structures in fostering adaptive outcomes.

At the same time, critics have noted that dominant, Western-derived resilience models are often applied uncritically in the Global South and other culturally diverse settings, where they may fail to capture indigenous coping strategies and culturally embedded strengths (6–8). Such models risk marginalizing local understandings and practices when they prioritize individual traits, skills, and symptom reduction over collective, moral, and relational dimensions of endurance. In response, scholars have called for culturally grounded resilience constructs, among which sumud — variously transliterated as sumud, sumood, and somoud, reflecting variation in Arabic-English transliteration rather than substantive conceptual differences (9–11) — has received growing attention. Across Arab cultural and social contexts, sumud denotes more than individual persistence; it refers to a relational and ethical orientation toward life, encompassing emotional anchoring, social cohesion, moral responsibility, and continuity of identity. It has been described as a way of living that integrates psychological endurance with communal solidarity and cultural continuity, often characterized as the art of enduring and sustaining dignity over time rather than overcoming adversity in a finite sense (12, 13). The gap this review addresses is a translational one: although sumud has been richly theorized across disciplines, its implications for psychotherapeutic practice remain scattered and had not previously been systematically synthesized.

Scholars have described sumud as involving both tangible and intangible dimensions. Tangible sumud refers to material infrastructures and practical supports that sustain daily life, such as educational institutions, healthcare services, community organizations, and informal social networks (14, 15). Intangible sumud encompasses belief systems, religious and spiritual values, cultural identity, social cohesion, and strong family support. Together, these intertwined dimensions function as protective resources that bolster individual and collective resilience under chronic adversity, aligning with social-ecological perspectives in resilience research (14–16). Conceptually, van Teeffelen and Biggs distinguish between passive or static sumud, emphasizing remaining rooted and maintaining continuity, and dynamic sumud, centered on everyday acts of moral agency and efforts to preserve dignity and coherence in the face of restrictive conditions (17, 18).

Religious and ethical traditions within the Arab world, particularly Islamic intellectual and spiritual traditions, have provided important frameworks for understanding and enacting everyday expressions of sumud. Values of endurance, patience, and moral perseverance are treated as ethical exemplars for sustaining hope, responsibility, and dignity during hardship (19). Early Islamic medical and ethical writings, such as treatises on the Sustenance of the Body and Soul, described processes such as internal psychological strategies and habituation to hardship, anticipating contemporary emphases on self-awareness, emotional regulation, flexibility, and mobilization of internal and external resources in the face of adversity (20, 21). These historical perspectives resonate with contemporary psychotherapeutic emphases on self-regulation, reflective capacity, and relational support, situating sumud within a long tradition of ethical and psychological reflection.

At the same time, sumud should not be understood as an exclusively religious notion, but as a broader cultural and ethical orientation that has evolved across Arab societies. It refers to the capacity to remain grounded in daily life, relationships, and social roles despite ongoing pressure, sustaining continuity of home, identity, and communal belonging (22).

Historically and contemporarily, this ethos is reflected across diverse cultural forms, including theatre, cinema, poetry, education, sport, and food practices, where sumud functions as a narrative, pedagogical, and expressive resource rather than a doctrinal concept (23–26). Collective cultural expressions such as group-based folkloric dance (dabke), artistic performance, literary production, and communal sporting or livelihood activities translate endurance into shared meaning, participation, and continuity. Everyday food practices and culinary traditions similarly operate as sites of cultural continuity and relational resilience, reinforcing belonging, memory, and social cohesion through shared routines and commensality (27).

Contemporary scholarship therefore conceptualizes sumud as a multidimensional form of resilience encompassing psychological, relational, cultural, and moral domains. Rather than denoting endurance alone, sumud reflects the capacity to sustain identity, dignity, language, values, and shared narratives over time, functioning as a relational process through which individuals and communities support one another (28). Empirical research identifies both tangible and intangible resources associated with sumud, including access to education and healthcare systems, collective social infrastructures, religious and cultural meaning systems, supportive interpersonal relationships, and individual psychological capacities such as self-regulation and purpose (16, 29, 30). Although findings on the direct relationship between hope and resilience are mixed (31, 32), hope has been linked to hardiness, self-efficacy, and the capacity to endure prolonged or potentially traumatic circumstances (33).

Taken together, these historical, ethical, and cultural strands suggest that sumud is best understood not as a single doctrinal construct but as a historically layered cultural framework shaped by spiritual traditions, social practices, embodied activities, and artistic expression. This framing is particularly relevant for psychiatry and psychotherapy, because it foregrounds resilience as embedded in meaning systems, moral orientation, and everyday cultural life rather than as an individual psychological skill alone. From a transcultural psychiatry perspective, sumud highlights how experiences of distress and endurance are organized through collective narratives, relational worlds, and institutional contexts, inviting clinicians to attend to these dimensions in assessment, formulation, and treatment planning.

Accordingly, this systematic review examines sumud as a framework for psychological resilience and evaluates its potential implications for psychotherapeutic practice across diverse cultural contexts. Specifically, we aim to synthesize how sumud has been conceptualized in interdisciplinary literatures as a pattern of endurance, meaning-making, and adaptive functioning, and to explore how these dimensions can inform the development of culturally responsive psychotherapeutic models. The review adopts a descriptive and analytical approach and does not advance political or ideological claims; studies are considered for their psychosocial and clinical insights rather than their political arguments. By positioning sumud as a conceptual resource rather than a political category or manualized intervention, we seek to clarify how collective and culturally grounded forms of resilience can inform psychotherapy in ways that are clinically useful, ethically attentive, and relevant to transcultural psychiatric practice.

## 2 Methods

### 2.1 Review design

This study is a systematic narrative review conducted in accordance with PRISMA 2020 guidance for systematic reviews without meta-analysis (34). The review aimed to synthesize existing evidence on sumud as a cultural construct of collective resilience and to examine its relevance to psychological resilience frameworks and psychotherapy models. A protocol specifying the review question, eligibility criteria, and analytical approach was developed *a priori* to ensure transparency and methodological consistency. This systematic review was registered with PROSPERO (CRD420261279052) on 5 February 2026 (35). A full PRISMA 2020 checklist is provided as **Supplementary Table 1**.

A systematic narrative review design, rather than a scoping review or qualitative evidence synthesis, was selected for three reasons. First, the review question required appraisal of a defined and pre-registered eligibility criterion (explicit engagement with sumud) rather than the broad, exploratory mapping of a field that a scoping review is designed to produce. Second, because the eligible literature spanned empirical studies, ethnographies, conceptual papers, and reviews, a qualitative evidence synthesis method built for a single study design (e.g., thematic synthesis of primary qualitative studies alone) was not well suited to the corpus; a narrative approach allowed heterogeneous evidence types to be integrated within a common analytical matrix while preserving their methodological distinctiveness. Third, a systematic — as opposed to purely narrative — search and screening process was retained to ensure transparency and reproducibility of study identification, consistent with PRISMA 2020 guidance for reviews where quantitative pooling is not appropriate (34).

### 2.2 Data sources and search strategy

A comprehensive literature search was conducted across four electronic databases: PubMed, Scopus, Web of Science, and Embase. The search was carried out between February and March 2026 and covered all publications available up to the search date, with no lower date limit applied, in order to capture the full body of sumud-related scholarship within contemporary resilience, mental health, and social science literatures. Searches were limited to English-language articles. Full-paper review commenced in March 2026, and full data extraction was finalized on 30 April 2026.

Database-specific search strategies combined controlled vocabulary terms (MeSH in PubMed, Emtree in Embase, and equivalent indexing terms in Scopus and Web of Science) with free-text keywords related to sumud and its spelling variants, psychological resilience and coping, cultural and collective contexts, and psychotherapy or mental health practice. Two complementary search strategies were developed to maximize retrieval: Strategy 1 targeted cultural and collective expressions of sumud (e.g., in the arts, education, and community life), and Strategy 2 focused on psychological resilience and therapeutic contexts. Both strategies were applied independently across all databases, and records were combined prior to deduplication and screening.

Search terms were organized around four core conceptual domains (1): sumud and its spelling variations; (2) psychological resilience and coping; (3) cultural and collective contexts; and (4) psychotherapy and mental health. The sumud domain included multiple transliterations (e.g., “sumud,” “sumoud,” “samoud,” “somoud”) searched primarily within titles and abstracts. Resilience-related terms combined free-text and controlled vocabulary, including “resilien*,” “psychological resilience,” “steadfastness,” “coping,” “coping strategies,” “endurance,” and “adaptation,” alongside relevant subject headings (e.g., Resilience, Psychological; Stress, Psychological; Adaptation, Psychological). Cultural and psychosocial contexts were captured using terms such as “culture*,” “cultural practices,” “collective identity,” “faith,” “religion,” “spirituality,” and terms referring to artistic and communal expressions, including art, poetry, theatre, cinema, dance, and sport. Therapeutic relevance was addressed using terms including “psychotherapy,” “counselling,” “therapy,” “mental health,” and “mental health services.” An example generic search string applied across databases was:

(“sumud” OR “sumoud” OR “samoud” OR “somoud”) AND (“resilien*” OR “psychological resilience” OR “steadfastness” OR “coping” OR “endurance” OR “adaptation”) AND (“culture*” OR “collective identity” OR “cultural practice*” OR “faith” OR “religion” OR “spirituality”) AND (“psychotherapy” OR “counselling” OR “therapy” OR “mental health”).

Full database-specific search strategies, including exact syntax and subject headings for each platform, are provided in **Supplementary Table 2**. To enhance completeness, reference lists of included studies and relevant reviews were manually screened to identify additional records.

### 2.3 Eligibility criteria

Studies were eligible for inclusion if they: (1) explicitly examined sumud (in any transliteration) as a cultural, psychological, or collective construct; (2) addressed themes related to resilience, coping, endurance, or psychological adaptation; and (3) included conceptual, psychosocial, or clinical discussion with relevance to mental health, wellbeing, or psychotherapy. Eligible publication types included peer-reviewed empirical studies (qualitative, quantitative, and mixed-methods), theoretical and conceptual papers, ethnographies, narrative analyses, and review articles discussing cultural, social, or therapeutic implications of sumud.

Studies were excluded if they: (1) did not explicitly reference sumud or its spelling variants; (2) focused exclusively on political, historical, or policy discourse without discernible psychosocial or mental health relevance; (3) were editorials, commentaries, conference abstracts without sufficient methodological detail, or dissertations; or (4) were not available in English. Studies with political content were retained where they provided substantive conceptualization of sumud in relation to resilience, meaning-making, or therapeutic practice. Restricting eligibility to English-language publications may have excluded relevant Arabic-, French-, or other language scholarship on sumud, including regional psychology and psychiatry journals and grey literature, and constitutes a potential source of language and publication bias; this limitation is revisited in Section 4.5.

### 2.4 Study selection

All records retrieved from database searches were exported and uploaded into Covidence systematic review software (36) for deduplication, reference management, and screening. The combined database search yielded 169 records (Scopus: n = 40; Web of Science: n = 35; PubMed: n = 14; Embase: n = 13; combined across both search strategies per database). An additional nine records were identified through manual searching, yielding 178 total raw records. After deduplication (67 entries removed), 111 unique records were imported into Covidence for screening.

Title and abstract screening and full-text screening were each conducted independently and in duplicate by two reviewers (D.A.E. and N.T.D.). Disagreements were resolved through discussion; where consensus could not be reached, a third reviewer (I.E.) was consulted and a final decision made by majority agreement. Following full-text review, 37 studies met the inclusion criteria and were included in the initial synthesis.

### 2.5 Data extraction and update search

Extraction was conducted independently and in duplicate by two reviewers (D.A.E. and N.T.D.) using a predefined analytical matrix developed *a priori* (**Supplementary Table 3**). A third reviewer (I.E.) independently verified a random 20% sample to provide an additional accuracy check. Disagreements were resolved through discussion and, where needed, adjudication by I.E.

For each included study, the matrix captured (1): publication characteristics (author, year, journal, country/region); (2) study design and methodology; (3) population and setting; (4) conceptualization of sumud; (5) dimensions of resilience and coping addressed; (6) cultural and collective elements; and (7) implications for mental health or psychotherapeutic practice. Extracted data were reviewed iteratively to identify recurring patterns and conceptual linkages relevant to collective resilience, cultural meaning-making, and psychotherapy. Summary characteristics and conceptual contributions of included studies are presented in **Table 1**.

**Table 1:** Characteristics and conceptual contributions of included studies on sumud (n = 39).

Study (Author, year)	Context / Setting	Study design	Population	Conceptualization of *sumud*	Key resilience dimensions	Mental health / psychotherapy relevance	Discipline
**Abudayya (2025)** (37)	Gaza	Qualitative	Civilians	Everyday coping under adversity	Meaning-making	Supportive, narrative therapy	Psychology/Mental Health
**Abuelaish & Yousufzai (2023)** (38)	Prolonged adversity	Conceptual	Populations	Moral, collective resilience	Dignity, hope	Community-centred MH models	Psychiatry / Global Mental Health
**Abushalha (2022)** (39)	Theatre	Performance analysis	Audiences	Unity through art	Symbolic cohesion	Cultural formulation	Humanities / Cultural & Performance Studies
**Al Jayousi & Nishide (2025)** (40)	NGOs	Qualitative	NGO staff	Organizational resilience	Knowledge-sharing	Organisational MH	Development Studies / Organisational Studies
**Aqtam et al. (2025)** (41)	Conflict-affected Palestinian communities	Qualitative	Not applicable	*Sumud* not explicitly theorised; resilience discussed primarily within global mental health and psychosocial intervention frameworks	Endurance, collective identity, everyday resistance, meaning-making	Culturally grounded, collective, and meaning-oriented care, with reduced emphasis on individualised recovery models	Psychiatry
**Atallah (2017)** (42)	Refugee communities	Qualitative, community-based	Families (intergenerational)	Collective, intergenerational endurance	Narrative, family, moral continuity	Supports family- and narrative-based therapy	Psychology/ transcultural Psychiatry
**Awayed-Bishara (2025)** (24)	Education	Linguistic ethnography	Youth	*Sumud* pedagogy	Language, identity	School-based MH relevance	Sociolinguistics
**Ayesh & Ben Hagai (2025)** (43)	Occupied settings	Qualitative	Adults	Recovery through persistence	Relational awareness	Context-aware psychotherapy	Psychology
**Bascuñan-Wiley (2019)** (27)	Chile diaspora	Ethnography	Families	Culinary *sumud*	Food, memory	Ritual in therapy	Anthropology / Cultural Studies / Diaspora Studies
**Bosqui et al. (2020)** (9)	Lebanon	Pilot intervention	Adolescents	“Somoud” as coping	Psychoeducation	Youth-friendly MH tools	Psychology
**Busse (2022)** (44)	Everyday life	Spatial analysis	Civilians	Spatial *sumud*	Routine, presence	Avoid pathologizing normality	Political Geography / International Relations
**Dabbagh et al. (2023)** (45)	Policy	Strategy paper	Children / adolescents	System-level resilience	Services, prevention	CAMH system design	Psychiatry
**Elkhalid et al. (2025)** (46)	Families	Narrative interviews	Parents / children	Storytelling as resilience	Intergenerational	Narrative family therapy	Communication Studies
**El-Shakry (2021)** (47)	Cultural theory	Conceptual	—	Existential endurance	Futurity, imagination	Existential Hypothesis	Postcolonial Studies
**Groten (2025)** (48)	Fishing communities	Ethnography	Workers	Livelihood endurance	Work, risk	Occupational MH	Anthropology / Cultural Geography / Middle East Studies
**Hamamra (2025)**	Youth culture	Qualitative	Youth	Survival pedagogy	Arts, testimony	Group youth interventions	Cultural Studies / Education / Theatre and Performance Studies
**Hamdonah & Joseph (2024)** (49)	Diaspora	Netnography	Youth / adults	Cultural continuity	Dance, embodiment	Group / expressive therapy	Media Studies / Cultural Studies / Diaspora Studies
**Hammad & Tribe (2021a)** (11)	Gaza	Qualitative	Adults	Adaptive coping as endurance	Meaning, faith, social ties	Strength-based clinical work	Psychology
**Hammad & Tribe (2021b)** (50)	Review	Narrative review	—	Culturally grounded resilience	Cultural, ethical	Critique of Western models	Psychology
**Harrold (2020)** (51)	Psychoanalytic	Conceptual	Communities	Ethical stance	Moral witnessing	Dignity-centered care	Psychology
**Isa (2023)** (52)	Urban precarity	Ethnographic essay	Residents	Presence amid insecurity	Identity, fear	Chronic stress formulation	Political Sociology / Anthropology / Middle East Studies
**Khodary et al. (2020)** (53)	Turkey / Iraq	Feminist qualitative	Women	Gendered endurance	Agency vs burden	Gender-sensitive care	Sociology
**Kteily-Hawa et al. (2020)** (54)	Gaza	Quantitative	Adults	Measured resilience	Social-ecological	Community resources matter	Public Health /
**Lax (2021)** (55)	Theatre	Case study	Artists	Creative endurance	Expression	Arts-based therapy	Theatre Studies / Cultural Studies
**Marie et al. (2016)** (29)	Health systems	Review	Population-level	Ecological resilience	Services, institutions	Contextualizes clinical care	Psychiatry
**Marie et al. (2017)** (30)	Mental health services	Qualitative	Nurses	Professional endurance	Ethical commitment, collegiality	Reflective practice, staff support	Psychiatry
**Marie et al. (2018)** (16)	Community ecology	Qualitative / theoretical	Communities	Social-ecological *sumud*	Relational, institutional	Systemic psychotherapy lens	Psychiatry
**Mason & Dajani (2019)** (37)	Golan Heights	Conceptual ethnography	Druze communities	Place-based endurance	Land, identity	Warns against individualization	Political studies
**Qudaih et al. (2025)** (56)	Health care	Survey	Physicians, nurses	Professional strain/endurance	Compassion, burnout	Staff support, supervision	Public health
**Roy (2016)** (57)	Urban studies	Theoretical	Urban residents	Everyday resilience	Space, class	Contextual MH thinking	postcolonial studies
**Ryan (2015)** (28)	Community settings	Feminist qualitative	Women	Everyday ethical endurance	Caregiving, relational responsibility	Warns against romanticizing endurance	Political Sociology
**Sazzad (2016)** (26)	Literature	Literary analysis	Cultural texts	Symbolic endurance	Poetry, language	Meaning-centred therapy	Other -Cultural Studies
**Scott et al. (2025)** (58)	Education / digital	Conceptual / practice	Learners, educators	Connected-learning *sumud*	Digital commons	Group / community therapy	Education / Digital Learning / Media Studies
**Taha et al. (2025)** (59)	Youth sport	Qualitative	Parkour practitioners	Embodied *sumud*	Movement, agency	Body-oriented therapy	Sociology
**Underhill (2022)** (60)	Visual art	Cultural analysis	Artists	Archival endurance	Memory, home	Narrative & trauma work	Visual art
**Veronese et al. (2021)** (39)	Health providers	Qualitative	Professionals	Meaning-based coherence	Ethics, identity	Existential psychotherapy	Transcultural psychiatry
**Wick (2008)** (61)	Reproductive health	Ethnography	Women / families	Reproductive endurance	Embodiment, care	Perinatal mental health	Cultural Psychiatry
Aqtam, Shouli & Ayed (2026)* (62)	Healthcare / West Bank (conflict setting)	Qualitative	Palestinian nurses	Professional endurance as moral witnessing; nurses as survivors sustaining care under systemic violence	Dual-role burden, ethical strain, moral commitment, cumulative professional stress	Staff support, reflective practice, supervision; cautions against romanticising professional endurance	Nursing / Transcultural Psychiatry
McMahon et al. (2026)* (63)	Gaza / occupied Palestinian territories	Conceptual / narrative review	Population-level	Sumud as culturally rooted collective protective factor; critique of Western models that individualise and depoliticise structurally produced suffering	Family cohesion, community solidarity, sumud; liberation psychology and decolonial mental health frameworks	Reframes distress as rational response to systemic violence; community-based, dignity-centred care; critiques individualistic psychiatric models	Psychiatry / Global Mental Health / Transcultural Psychiatry

Cells shaded and marked with an asterisk (*) in the Discipline column indicate a discipline label standardized for consistency (e.g., “Psychology/Mental Health” consolidated to “Psychology”; multi-discipline entries shortened to the primary discipline). † Studies added following the June 2026 update search, shaded for visibility, consistent with their separate identification in the PRISMA 2020 flow diagram (**Figure 1**).

To ensure that no eligible publications had appeared in the interval between the completion of data extraction and submission of this manuscript, a rapid update search was conducted on 1 June 2026 across all four databases using the identical search strategies applied in the original search. Two additional studies meeting the predefined eligibility criteria were identified: Aqtam, Shouli & Ayed (62), and McMahon et al. (63). Both studies were assessed for eligibility independently and in duplicate, extracted using the same predefined matrix, and verified by a third reviewer, in accordance with the procedures applied to all originally included studies. Their incorporation brings the total number of studies included in the final synthesis to 39. The study selection process is summarized in the PRISMA 2020 flow diagram (**Figure 1**).

**Figure 1:**
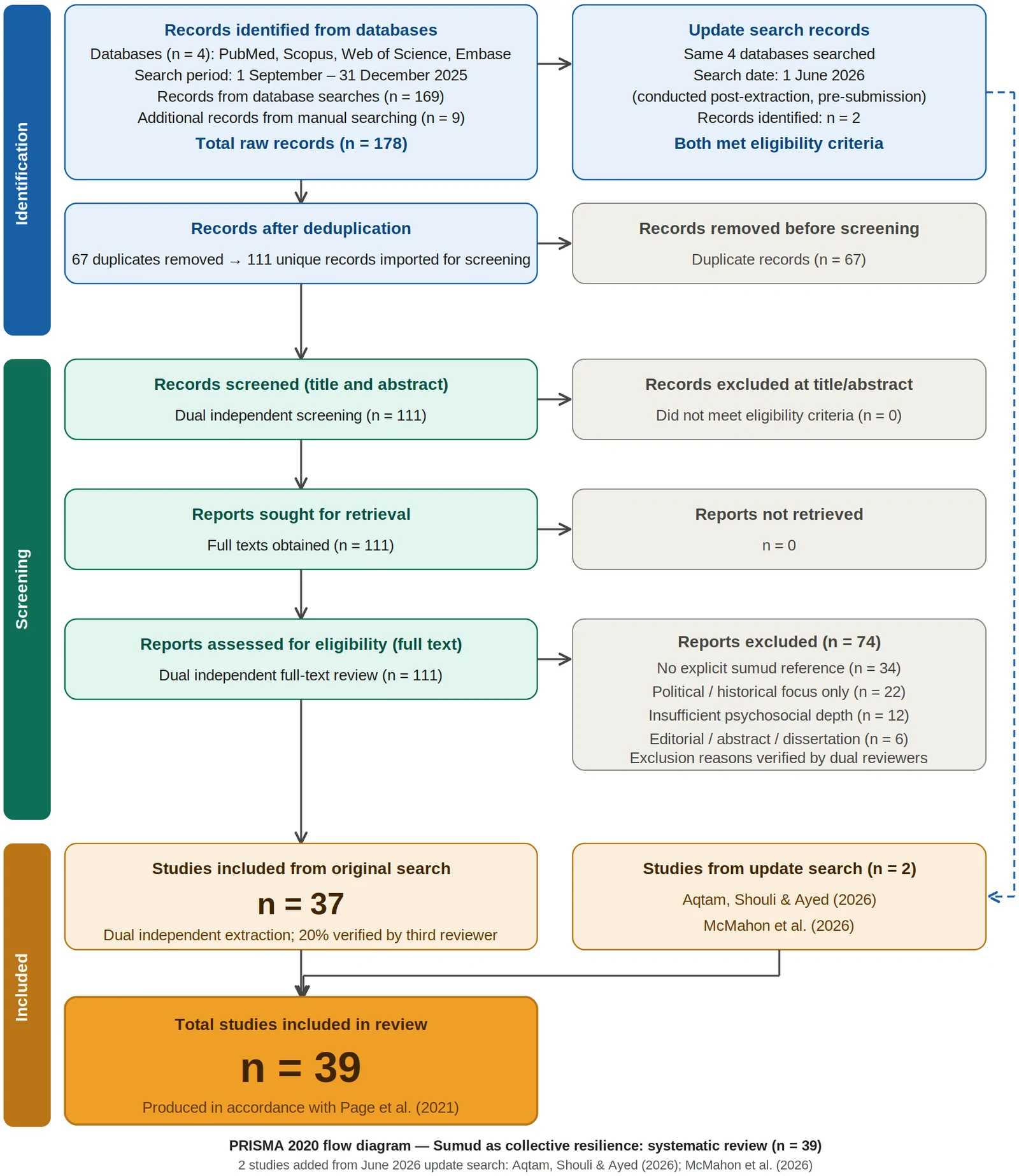
PRISMA flowchart showing included studies. PRISMA 2020 flow diagram illustrating the systematic literature search and study selection process. The diagram details the number of records identified across databases (PubMed, Scopus, Web of Science, Embase) and through manual searching; records screened by title and abstract; full-text articles assessed for eligibility; and studies included in the final synthesis (n = 39), with reasons for exclusion at each stage. Produced in accordance with Page et al. (2021).

### 2.6 Quality appraisal

Given the methodological heterogeneity of the included literature — encompassing qualitative, ethnographic, conceptual, and policy-oriented work alongside limited quantitative studies — formal risk-of-bias tools designed for intervention or observational trials were not applied. Instead, studies were appraised narratively using the analytical matrix, with attention to methodological clarity, transparency of data collection and analysis, conceptual coherence, and relevance to the review objectives. This approach aligns with PRISMA 2020 recommendations for reviews where quantitative synthesis and standardized bias assessment are not appropriate. Methodological clarity was operationalized as explicit reporting of sampling or case-selection rationale, data collection procedures, and analytic approach. Conceptual coherence was operationalized as the internal consistency between a study’s stated conceptualization of sumud and the evidence or argument offered in support of it, together with the clarity with which resilience-related constructs were defined and distinguished from related terms (see **Table 2**). Both criteria were assessed by the same two reviewers responsible for data extraction (D.A.E. and N.T.D.) and recorded as brief narrative notes within the extraction matrix rather than as a numeric score; disagreements in appraisal were resolved through the same discussion-and-adjudication procedure described in Section 2.5.

**Table 2:** Conceptual distinctions between *sumud* and related constructs.

Concept	How it differs from *sumud*
Coping	More technical, often individual and strategy-based
Resilience	Presupposes recovery and restoration of functioning
Stoicism	Primarily philosophical and individual
Resignation	Passive, lacks agency and intentionality
Endurance	Does not sufficiently account for meaning, identity, and sociopolitical context

For conceptual and theoretical papers specifically, which cannot be appraised against criteria designed for empirical data collection, the matrix instead recorded: (i) whether the paper’s central claims about sumud were grounded in cited primary sources, fieldwork, or textual evidence rather than assertion alone; (ii) whether the paper’s argument was internally consistent and clearly distinguished sumud from adjacent constructs (see **Table 2**); and (iii) whether the paper’s scope and claims were proportionate to the evidence presented. This distinction between empirical and conceptual appraisal criteria is now stated explicitly to improve transparency for readers assessing the review’s methodological rigor.

## 3 Results

### 3.1 Overview of included studies

The final synthesis comprised 39 studies spanning psychology, psychiatry, public and global mental health, nursing, anthropology, education, development studies, geography, and the humanities. Study designs were predominantly qualitative, ethnographic, narrative, and conceptual, with fewer quantitative or mixed-methods studies and a small number of systematic or narrative reviews. This methodological profile reflects the exploratory and context-sensitive nature of research on sumud and collective resilience in prolonged adversity.

Populations studied were diverse and included civilians and families, women, children and adolescents, healthcare professionals, NGO workers, educators, cultural practitioners, artists, athletes, diaspora communities, and policy- or system-level stakeholders. Most studies were conducted in Palestinian contexts, with additional work in Lebanon, Turkey, Iraq, Syria, and diaspora settings such as Chile, enabling comparative and transnational perspectives on endurance and resilience. The most recent studies, including those by Awayed-Bishara (24) and Scott et al. (58), extended sumud scholarship into educational and learning contexts. Key characteristics and conceptual contributions of the included studies are summarized in **Table 1**. Terminology in the Discipline column of **Table 1** has been standardized across entries (see table note) to improve consistency and readability.

### 3.2 Conceptualizations and levels of resilience

Across the corpus, sumud was consistently conceptualized not as a fixed individual trait but as a multilevel, relational, and collective form of resilience. The most frequently identified levels were collective, relational, and community-based, typically embedded within families, professional groups, cultural practices, or organizational structures. Several studies highlighted intergenerational resilience, emphasizing the transmission of endurance through storytelling, caregiving, shared values, and moral continuity across generations (16, 28, 42).

Individual resilience appeared more prominently in quantitative or clinically oriented work but was almost always situated within broader social, cultural, and ecological contexts rather than treated as a standalone capacity (29, 54). Sumud was rarely framed as passive endurance; instead, it was described as an active, ethically informed orientation toward life, encompassing dignity, responsibility toward others, and commitment to sustaining everyday roles, identities, and routines over time (28, 61). Several authors warned against romanticizing endurance or equating sumud with unqualified resilience, noting tensions between agency, burden, and cumulative strain, particularly in gendered analyses (28, 64).

### 3.3 Dimensions of resilience and coping

Thematic analysis identified multiple interrelated dimensions of resilience and coping associated with sumud.

At the psychological level, resilience was linked to meaning-making, emotional regulation, self-awareness, hope, and perceived self-efficacy, but these capacities were consistently described as relationally scaffolded rather than purely intrapsychic (30, 33, 50). Hope emerged as an important, though not uniformly defined, dimension: while quantitative studies reported mixed findings on its direct association with resilience (31, 32), qualitative accounts underscored hope as embedded in shared narratives, ethical commitments, and future-oriented responsibilities.

At the relational and social level, sumud was enacted through caregiving, mutual support, intergenerational transmission of memory, and collective moral frameworks. Family systems and close social networks were described as primary sites through which resilience was sustained and transmitted, particularly for women, children, and older adults (42, 46, 61). Studies focused on women highlighted everyday responsibilities, caregiving labor, and cultural transmission as central expressions of sumud, while also drawing attention to ethical tensions and cumulative psychological and social burdens (28, 64).

At the cultural and embodied level, many studies documented resilience as expressed through arts, sports, and everyday bodily practices, including theatre, music, poetry, visual art, dance (notably dabke), parkour, fishing, communal food practices, and archival or memory work (26, 27, 48, 49, 55, 60). These activities functioned as collective psychosocial resources that supported continuity, identity, and belonging through participation, rhythm, repetition, and shared expression, and they were generally described as integral components of lived resilience rather than as ancillary coping techniques.

At the ecological and institutional level, resilience was linked to access to education, healthcare systems, community organizations, and informal social infrastructures. Organizational and professional studies described sumud as sustained through shared purpose, ethical commitment, collegial support, and collective responsibility in healthcare, NGO, and educational settings (29, 30, 40, 45). Policy-oriented work conceptualized sumud at the system level, emphasizing child and adolescent mental health services, prevention, and service design in contexts of chronic adversity (45). These dimensions are visualized in the socio-ecological framework presented in **Figure 2**.

**Figure 2:**
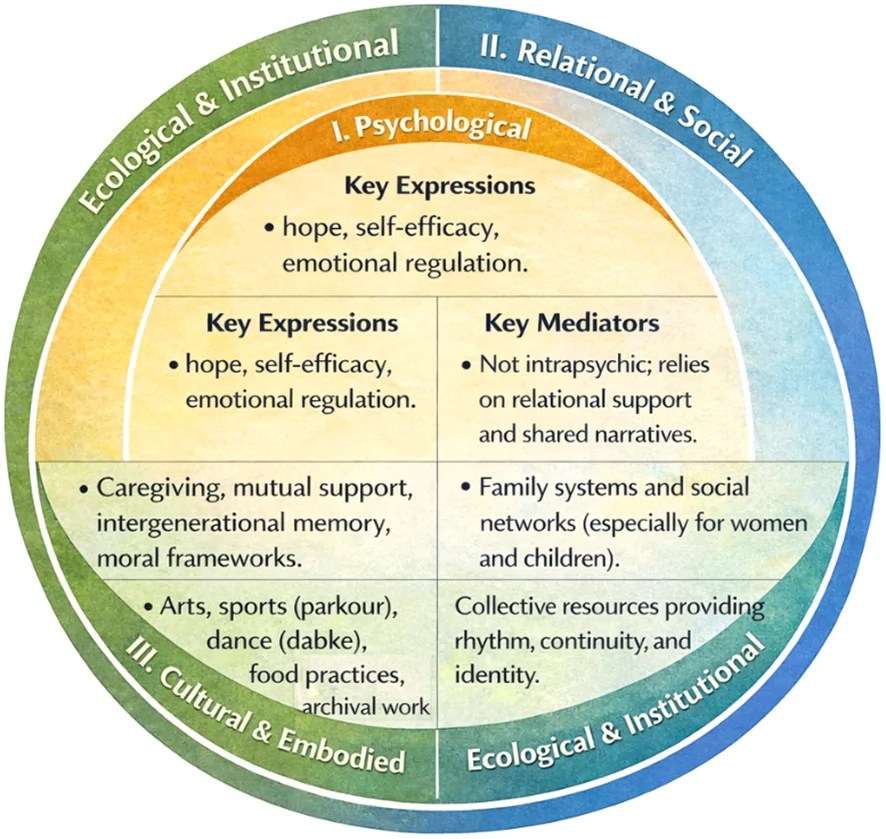
Socio-ecological framework of sumud as collective resilience, illustrating the interrelated dimensions identified across included studies: psychological (meaning-making, emotional regulation, hope, self-efficacy); relational and social (family systems, caregiving, intergenerational transmission, mutual support networks); cultural and embodied (arts, dance, sport, food practices, embodied routines); and ecological and institutional (education, health-care systems, community organizations, NGOs, policy structures). The framework positions sumud as a multilevel, relationally embedded process rather than an individual psychological trait, consistent with social-ecological models of resilience.

### 3.4 Gender, developmental, and educational dimensions

Gender and developmental perspectives were unevenly represented but conceptually important. A subset of studies explicitly centered women’s experiences, conceptualizing resilience through caregiving, reproductive labor, cultural transmission, and everyday responsibility, and highlighting the coexistence of agency and burden (28, 61, 64). These analyses cautioned against idealizing sumud in ways that obscure distress, structural constraints, or the unequal distribution of labor within households and communities.

Youth resilience was described as emerging through family narratives, educational environments, peer relationships, and opportunities for creative and expressive participation (9, 42, 46). Educational and pedagogical contexts, both formal and informal, were identified as important sites for resilience development, particularly through storytelling, arts-based learning, psychoeducation, and value-based dialogue (10, 24, 58). Recent work by Awayed-Bishara (24) and Scott et al. (58) strengthened this dimension by explicitly conceptualizing sumud within educational, linguistic, and digital learning spaces.

### 3.5 Mental health and psychotherapy-relevant findings

Studies situated within mental health disciplines converged on a social-ecological understanding of resilience that integrates individual, relational, community, and institutional factors. Healthcare professionals and mental health workers frequently described resilience as tied to professional identity, ethical responsibility, and sustained caregiving under high demand, with sumud invoked to articulate meanings of endurance, commitment, and moral coherence in practice (30, 56, 65). Quantitative findings suggested that resilience could be maintained despite substantial stress exposure but was strongly associated with social connectedness, education, and community resources rather than individual characteristics alone (29, 54).

Across the synthesis, clear implications for psychotherapy emerged. First, individualized and decontextualized psychotherapeutic models appeared insufficient in settings characterized by chronic or structural adversity, where distress and coping are organized through collective histories, family roles, and community practices (16, 50). Psychotherapy alignment with sumud was strongest in studies emphasizing narrative, family-based, group, and community-oriented relational worlds (9, 42, 46, 65). For children and adolescents, developmentally appropriate, creative, and psychoeducational modalities involving arts, storytelling, and play were highlighted as particularly congruent with resilience processes (9, 10, 59).

Second, several studies involving professionals underscored the importance of reflective spaces, peer support, and interventions addressing ethical strain and cumulative stress, suggesting that sumud may also inform staff support and supervision models in mental health and related services (30, 56, 65). A fuller discussion of this cautionary evidence, and its implication that sumud should not be romanticized in staff-support or supervision models, is developed in Section 4.1.

### 3.6 Methodological patterns and gaps

Methodologically, the dominance of qualitative, ethnographic, and conceptual work allowed for rich contextual and cultural analysis but limited causal inference and systematic outcome evaluation. Across studies, there was a notable absence of rigorously evaluated psychotherapeutic interventions explicitly grounded in sumud. Longitudinal designs, mixed-methods approaches, and quantitative outcome measures were rare. Children, adolescents, and older adults were underrepresented outside policy or review-based work, and gender analyses were inconsistently applied. Geographic coverage was concentrated in Palestinian settings, with limited exploration of how sumud-like frameworks might appear or be adapted in other cultural settings. These gaps inform the subsequent discussion on research priorities and implications for transcultural psychiatric practice.

**Table 3** presents a structured comparison of resilience and sumud along key conceptual dimensions. **Table 2** presents conceptual distinctions between sumud and related constructs.

**Table 3:** Structured comparison between resilience and *sumud*, alongside distinctions from related constructs.

Dimension	Resilience (dominant model)	*Sumud*
Core orientation	Individual adaptation	Collective endurance
Temporal dynamic	Return to equilibrium	Persistence in the absence of equilibrium
Functional capacity	Capacity to bounce back	Capacity to hold on / endure
Temporal focus	Future-oriented	Grounded in the present
Assumption about change	Change is possible	Change is blocked

## 4 Discussion

This systematic review synthesized how sumud and resilience have been conceptualized across interdisciplinary literatures, with a particular focus on their relevance for mental health and psychotherapy. Across 39 included studies, a clear and consistent pattern emerged: sumud was not conceptualized as an individual psychological trait but as a collective, relational form of resilience sustained through families, communities, cultural practices, professional roles, and institutional arrangements. This pattern highlights the cultural specificity of resilience conceptualizations and exposes the limitations of dominant Western definitions, which have historically prioritized individual adaptation, personal coping skills, and symptom-focused recovery (66, 67). More recent scholarship has explicitly critiqued this individualization, arguing that such models insufficiently capture resilience as it is lived and sustained in diverse cultural settings (6, 68, 69). Notably, despite the conceptual richness of this literature, there remains a clear gap in the explicit translation of sumud into psychotherapeutic theory, intervention design, and clinical practice, an absence this review seeks to identify and address.

### 4.1 Reconceptualizing resilience: from individual to collective

Dominant resilience models in psychology and psychiatry have traditionally conceptualized resilience as an individual-level capacity, emphasizing personal traits, coping skills, and symptom reduction in response to adversity (66, 67, 70). In contrast, the findings of this synthesis consistently challenge this framing. Across disciplines and methodologies, sumud was treated as a collective form of resilience that is relationally produced, ethically grounded, and sustained through shared social worlds, including families, communities, cultural practices, and institutions (11, 16, 42).

Within the reviewed literature, sumud is fundamentally relational, emerging through family bonds, intergenerational transmission, professional solidarity, and communal participation (16, 42). It is explicitly ethical, anchored in moral responsibility, care for others, and the preservation of dignity across time, particularly in caregiving, professional, and civic roles (28, 51). In addition, sumud is profoundly meaning-oriented and existential, providing frameworks through which individuals and communities interpret suffering, sustain hope, and affirm continuity of self and collective identity (46, 47).

Religious and spiritual dimensions constitute an important component of this collective resilience. Several studies describe faith, spirituality, and religious practice as shared meaning systems that support endurance, ethical commitment, and existential coherence over time, without reducing resilience to individual religiosity or private belief (11, 30). One study explicitly situates sumud within a Muslim-Christian context, highlighting interreligious coexistence and shared moral values as collective resources for resilience embedded in educational and civic life (71). In this sense, religion functions not primarily as a coping strategy but as a collective existential orientation that structures meaning, belonging, moral responsibility, and continuity in the face of prolonged adversity, consistent with existential perspectives in psychiatry and psychotherapy (72, 73).

Crucially, sumud is embedded within social, cultural, and institutional contexts, including education, health care, arts, sports, and community organizations, rather than residing solely within individuals. This understanding aligns closely with transcultural psychiatry, which emphasizes culturally mediated meanings of distress and resilience, and with social-ecological models of resilience, which conceptualize wellbeing as emerging from interactions between individuals, relationships, communities, and institutions (11, 65). Viewed through these frameworks, sumud represents a conceptual shift from resilience as personal adaptation toward resilience as a collectively organized, ethically and spiritually grounded, and existentially meaningful process of living with and through ongoing adversity. Consistent with the Results (Section 3.5), several studies cautioned specifically against romanticizing this endurance: framing sumud purely as strength risks overlooking exhaustion, burnout, or constrained choice, particularly for women and other marginalized groups (28, 64, 74). This caution carries a direct clinical implication, elaborated in Section 4.4, that dignity-centered care must remain attentive to the costs of endurance rather than treating sumud as an unqualified therapeutic asset.

These findings raise the question of what sumud contributes beyond adjacent resilience constructs already established in the literature, including social-ecological resilience, family resilience, community resilience, and liberation psychology. Three features distinguish sumud within this synthesis. First, whereas social-ecological and family resilience models typically retain an implicit assumption that adversity is time-limited and that adaptation moves toward eventual stability, sumud as described across the reviewed studies explicitly presupposes conditions under which resolution or recovery is not imminently available; it is oriented toward sustaining function and identity within adversity rather than toward a return to a prior equilibrium (see **Table 3**). Second, sumud foregrounds the preservation of dignity and identity continuity as ends in themselves, not merely as instrumental means to symptom reduction or functional adaptation — several studies described enduring with dignity as the substantive goal of sumud, independent of whether material circumstances improved (13, 28, 51). Third, sumud carries an explicit ethical dimension of collective responsibility that is less consistently foregrounded in social-ecological or family resilience frameworks: enduring is framed not only as self-preservation but as a moral obligation toward family, community, and cultural continuity (28, 51, 71). Liberation psychology shares sumud’s attention to structural adversity and its critique of individualizing frameworks, but liberation psychology is oriented primarily toward conscientization and collective action aimed at structural change, whereas sumud, as synthesized here, encompasses a broader repertoire that includes non-confrontational, everyday, and embodied forms of endurance that do not necessarily aim at transforming the structural conditions themselves. Clarifying these distinctions positions sumud as a conceptually distinct, rather than redundant, addition to the resilience literature.

### 4.2 Embodied, cultural, and communal expressions of sumud

A substantial body of the literature conceptualized sumud through embodied, cultural, and communal practices, including theatre, music, poetry, visual art, dance, sport, livelihood activities, and everyday bodily routines. Rather than framing these practices as individual coping strategies, studies consistently positioned them as collective psychosocial processes that sustain continuity, identity, and shared meaning over time. Theatre, music, and poetry were described as settings for collective narration and emotional expression, where endurance is enacted through shared performance, memory, and voice (10, 26, 28, 55). Visual and archival art similarly functioned as communal practices of remembrance and symbolic homemaking, supporting continuity and collective presence through aesthetic form (60).

Embodied and movement-based practices, including dabke dance, parkour, fishing, and everyday bodily engagement with place, were particularly salient in articulating sumud as lived and enacted through the body (48, 49, 59). These practices foreground participation, rhythm, repetition, and spatial orientation, reinforcing sumud as a shared and performative process rather than an internal psychological state. In this literature, dabke was conceptualized not merely as cultural expression but as a re-embodied articulation of attachment and continuity, in which collective movement, synchrony, and circular formation enact rootedness, belonging, and connection to place, even in contexts characterized by displacement or mobility (49).

Applied to the present synthesis, embodied cultural practices associated with sumud can be understood as relational and existential processes that support dignity, coherence, and continuity through participation and shared meaning, supporting psychological and social continuity not primarily by reducing symptoms but by anchoring individuals within shared rhythms, relationships, and narratives (75, 76).

### 4.3 Developmental and gendered dimensions

Across the reviewed literature, sumud was frequently articulated as a developmental and intergenerational process, transmitted through storytelling, memory, caregiving practices, and shared moral frameworks. Family-based and narrative studies highlighted how intergenerational transmission of experiences, values, and historical memory served as a key mechanism sustaining continuity and endurance over time (42, 46). Storytelling served not only as a means of preserving collective memory but also as a relational process that scaffolded meaning, identity, and emotional regulation across generations.

Studies focusing on children and adolescents consistently framed resilience as relational and developmentally situated rather than as an individual capacity for self-reliance. Youth resilience was described as emerging through supportive relationships with caregivers, peers, educators, and community members, as well as through opportunities for narrative expression, creative engagement, and participation in culturally meaningful activities (9, 10). These studies emphasized that resilience in younger populations was shaped by developmental scaffolding, including protection, guidance, and shared meaning-making, rather than by expectations of premature autonomy or emotional self-sufficiency (53).

Studies centering women’s experiences described sumud as enacted through community life through caregiving, emotional labor, cultural transmission, and everyday practical responsibility, often sustained through intergenerational and extended-family networks (28, 61). At the same time, this literature explicitly cautioned against romanticizing endurance, noting that gendered resilience often involved significant cumulative burden, constrained choice, and ethical tension. Feminist and clinical perspectives underscored the importance of recognizing both agency and vulnerability, warning that uncritical celebration of endurance risks obscuring distress, silencing suffering, and reinforcing unequal expectations of care and sacrifice (16, 77).

### 4.4 Implications for psychotherapy practice

Building on the reconceptualization of resilience developed in Section 4.1, individualized, symptom-focused psychotherapeutic models are insufficient when resilience is understood as a collective, relational, and meaning-oriented process. While such approaches may be effective for discrete stressors, they can be limited in contexts characterized by chronic and cumulative adversity, where distress is embedded within enduring relational, social, and moral worlds.

Translating sumud into psychotherapy principles aligns closely with relational, systemic, and meaning-centered traditions within Western psychotherapy. First, the emphasis on continuity, narrative, and shared meaning resonates with narrative and family-based approaches, which conceptualize psychological wellbeing as emerging through relational stories, identity construction, and intergenerational meaning-making (78, 79). Second, the collective nature of sumud supports the relevance of group and community-based interventions, which emphasize that psychological change occurs through interpersonal processes, collective witnessing, and the normalization of shared experience (80). Third, the prominence of embodied, cultural, and expressive practices aligns with arts-based and body-oriented psychotherapies, which conceptualize healing as emerging through embodied participation, affective expression, and relational attunement rather than verbal insight alone (81, 82). Across these modalities, a unifying implication is the importance of ethical witnessing and dignity-centered care, well established in existential and relational psychotherapy (72, 73).

Developmentally, these implications vary across populations. For children and adolescents, developmentally sensitive and family-inclusive interventions are supported by the finding that resilience is relationally scaffolded through caregivers, families, and social environments (83, 84). For families, systemic and relational therapies provide frameworks for working with shared narratives and intergenerational continuity. For professionals and caregivers, reflective practice and group-based support address ethical strain and cumulative stress more effectively than individual stress-management approaches (85, 86).

These implications are particularly salient in the context of ongoing conflicts, displacement, and humanitarian crises affecting parts of the Middle East and Gulf region. In such contexts, adversity is often prolonged, collective, and structurally embedded, shaping psychological experiences in ways that extend beyond individual stress responses. Frameworks such as sumud may offer culturally grounded resources for sustaining psychological continuity, dignity, and meaning under conditions of persistent uncertainty and instability, and clinicians working in conflict-affected or politically volatile environments may find sumud a particularly relevant conceptual resource for clinical formulation and therapeutic stance.

Beyond these general orientations, sumud can inform specific, concrete points in the clinical pathway. In cultural assessment, clinicians can routinely ask about sources of endurance, collective belonging, and moral obligation, rather than confining assessment to individual symptom inventories, to surface relational and cultural resources that standard intake tools may miss. In case formulation, distress presenting as persistence of low mood or anxiety under chronic, unresolved adversity can be provisionally understood as a rational response to sustained structural strain rather than solely as pathology, without foreclosing formal diagnosis where clinically indicated; this reframing affects how goals of treatment are negotiated with the patient. Regarding diagnosis, clinicians are encouraged to distinguish presentations of endurance and moral commitment from presentations of depressive withdrawal or anhedonia, since the two can be phenomenologically similar but carry different treatment implications. For the therapeutic alliance, explicitly naming and validating sumud as a recognized cultural resource, rather than treating the patient’s endurance as mere coping to be clinically neutral about, can strengthen engagement and reduce the risk that patients experience Western-derived frameworks as dismissive of their moral and relational world. In intervention planning, sumud-informed practice can be integrated into existing modalities rather than requiring a new manualized protocol: narrative therapy can be adapted to elicit and re-author sumud-related life stories (78, 79); family systems therapy can map intergenerational transmission of endurance and identify where caregiving burden has become unsustainable (42, 46); acceptance-based therapies (e.g., Acceptance and Commitment Therapy) can draw on sumud’s orientation toward sustaining valued action under conditions that cannot be immediately changed, rather than pursuing symptom elimination; and culturally informed cognitive behavioral therapy can incorporate sumud-consistent meaning-making and dignity-preserving goals alongside standard cognitive and behavioral techniques, adjusting rather than replacing the model’s structure.

### 4.5 Methodological strengths and limitations

This review has several methodological strengths. Its interdisciplinary scope brings together evidence from psychiatry, psychology, public and global mental health, education, the social sciences, sports, the arts, and the humanities, allowing for a comprehensive examination of sumud that would not be possible within a single disciplinary lens. The reviewed literature demonstrates considerable cultural depth, privileging meaning-making, moral values, historical continuity, and shared practices over decontextualized individual traits. Many studies adopt an ecological framing, situating resilience across multiple levels.

At the same time, important limitations were identified. There is a lack of intervention-based studies that translate collective or relational conceptualizations of resilience into empirically evaluated psychotherapeutic or psychosocial interventions. Longitudinal research is limited, restricting understanding of how collective resilience processes evolve over time. Outcome measures are often poorly aligned with collective resilience constructs, relying instead on individual symptom scales. Geographic coverage was concentrated in Palestinian settings, with limited exploration of how sumud-like frameworks might appear or be adapted in other cultural settings.

Restricting eligibility to English-language publications, as noted in Section 2.3, may further have excluded relevant Arabic-, French-, or Hebrew-language scholarship, particularly regional psychology and psychiatry journals and grey literature, introducing a potential source of language and publication bias alongside the geographic concentration noted above.

Addressing these gaps will require mixed-methods designs that integrate qualitative depth with quantitative rigor, as well as community-engaged and participatory research approaches. More specifically, we recommend: (i) development and psychometric validation of a sumud-specific or collective-resilience outcome measure suitable for use alongside, rather than as a substitute for, individual symptom scales; (ii) longitudinal cohort designs following families or professional cohorts (e.g., community mental health nurses, as in Marie et al., 2017) across periods of acute versus chronic adversity to examine how sumud-related processes change over time; (iii) intervention-development studies that pilot narrative, family-based, or arts-informed approaches explicitly grounded in sumud with pre-registered outcome measures, directly addressing the absence of evaluated interventions noted in Section 3.6; and (iv) replication of the socio-ecological framework presented in non-Palestinian Arab and diaspora settings to test its transferability.

### 4.6 Clinical and conceptual contributions

This review makes several clinical and conceptual contributions. Conceptually, it reframes resilience as a collective phenomenon, challenging dominant Western models that locate resilience primarily within individual traits. By synthesizing evidence across disciplines, the review demonstrates that resilience can be more accurately understood as emerging through relationships, shared meanings, ethical commitments, and embodied participation in social life. Clinically, the review bridges social-science scholarship and psychotherapy, showing how cultural, relational, and embodied practices are directly relevant to mental health practice.

Importantly, this review positions sumud not as a technique, intervention, or manualized treatment, but as a conceptual resource that can inform clinical thinking, formulation, and therapeutic stance. As such, sumud offers clinicians a culturally grounded lens through which to understand endurance, continuity, and dignity in contexts of prolonged adversity, without prescribing a specific therapeutic method. This distinction is critical to avoid the instrumentalization of culturally rooted concepts and to preserve their ethical and relational significance.

### 4.7 Situating sumud within transcultural psychiatry and global mental health scholarship

The present synthesis is consistent with, and extends, foundational critiques within transcultural psychiatry of the uncritical export of Western diagnostic and therapeutic categories. Kleinman’s argument that suffering and healing are shaped by local moral worlds and explanatory models, rather than existing as culture-free biomedical facts, anticipates this review’s central claim that sumud constitutes a locally meaningful idiom of distress and endurance rather than a deficient or informal version of resilience (87). Kirmayer’s critique of the ‘new cross-cultural psychiatry’ — the caution that culture is too often treated as a superficial modifier of otherwise universal psychiatric categories rather than as constitutive of how distress is experienced and expressed — is directly applicable to sumud, which this review has shown to be constitutive of, rather than incidental to, how endurance and coping are lived in Arab contexts (87). Summerfield’s critique of the medicalization of human suffering under conditions of war and political violence similarly supports the caution, developed in Section 4.8 below, against treating chronic adversity primarily as a psychiatric condition to be resolved through individual-level intervention (88).

Situating sumud alongside other culturally grounded, non-Western resilience and personhood concepts further clarifies both its shared features and its specificity. Ubuntu, the Southern African ethic captured in the phrase ‘I am because we are,’ shares with sumud a relational and collective conception of personhood and an ethic of mutual obligation, but is oriented primarily toward restorative justice, reconciliation, and communal harmony rather than toward endurance under conditions of unresolved, ongoing structural adversity (89). Buen Vivir, the Andean concept of collective wellbeing in balance with community and nature, shares sumud’s rejection of individualistic, growth- or recovery-oriented models of wellbeing, but is grounded in a cosmological relationship to land and nature that is less central to sumud’s primarily social, ethical, and political grounding (90). Kapwa, the Filipino concept of shared identity and interconnectedness with others, resembles sumud’s emphasis on relational continuity of self, but has been developed primarily within decolonial Filipino psychology as a framework for interpersonal ethics rather than for enduring chronic political adversity specifically (91). This comparative positioning suggests that sumud belongs to a broader family of non-Western, collectively oriented personhood and resilience concepts, while retaining a distinctive emphasis — elaborated in Section 4.1 — on dignified endurance under conditions where resolution is not immediately available.

### 4.8 Critical reflections on the limits of sumud

Although this review positions sumud as a valuable resource for transcultural psychiatric practice, the concept warrants sustained critical scrutiny rather than uncritical clinical adoption. Several risks emerge from a closer reading of the included literature. First, an uncritical framing of sumud risks normalizing suffering: if enduring hardship is valorized as a cultural and moral good, patients and communities may come to interpret psychological distress as an expected or even virtuous cost of adversity, delaying recognition of clinically significant conditions that warrant treatment (28, 64). Second, and relatedly, sumud may reinforce expectations of self-sacrifice, particularly for women and caregivers, whose endurance is frequently the mechanism through which family and community sumud is sustained; several studies documented this burden explicitly, but the broader discourse around sumud does not always make the unequal distribution of this labor visible (28, 61, 64). Third, sumud may discourage help-seeking: patients and communities that understand psychological distress through a framework of moral endurance may be less likely to view formal mental health services as relevant or necessary, particularly where such services are unfamiliar, stigmatized, or scarce (30, 56). Fourth, and most fundamentally, sumud risks being read by clinicians, institutions, or policymakers as an individual or communal psychological strength rather than as a response to structural injustice, thereby obscuring the political and material conditions that make endurance necessary in the first place; several authors in this synthesis, including the update-search study by McMahon et al. (63), explicitly warned against this depoliticizing reading (6, 11, 68, 88). Taken together, these tensions suggest that sumud should be approached by clinicians as a resource to be explored collaboratively with patients rather than assumed, and that its clinical use should be accompanied by explicit attention to whether endurance is being sustained at an unsustainable personal or relational cost, and to the structural conditions underlying the adversity being endured.

## 5 Conclusion

This review’s contribution is threefold and, in combination, novel relative to prior single-discipline treatments of sumud, such as Hammad & Tribe’s (50) literature review, which focused specifically on the occupied Palestinian territories and conflict-related coping. First, its interdisciplinary scope — spanning psychiatry, psychology, nursing, education, the arts, and the social sciences — allowed the socio-ecological framework presented in **Figure 2** to be constructed from evidence that no single-discipline review had previously integrated. Second, it explicitly translates sumud into concrete psychotherapeutic modalities (narrative, family-based, group, and arts-based approaches) rather than treating it solely as a descriptive cultural or political construct. Third, it identifies a specific and previously unarticulated research gap — the near-total absence of rigorously evaluated, sumud-informed psychotherapeutic interventions — which we position as the field’s principal priority going forward.

As established in Sections 3 and 4.1, sumud is best understood as a collective, relational, and meaning-oriented form of resilience oriented toward dignified endurance rather than recovery from discrete stressors — and, as Sections 4.7 and 4.8 make clear, this endurance carries both cultural value and ethical cost. The conceptual and clinical insights derived from this synthesis have relevance for psychotherapy practice in culturally collectivist and family-oriented contexts, including many Arab societies, as well as for transcultural psychiatry more broadly.

For clinicians, this review underscores the importance of engaging with patients’ relational worlds, cultural narratives, and collective practices as resources for endurance and meaning-making, while remaining attentive to the ethical costs and cumulative burdens that sustained endurance can impose, particularly for women, caregivers, and those in professional roles. For researchers, the review identifies a substantial gap in rigorously evaluated, sumud-informed psychotherapeutic interventions. Future studies should prioritize longitudinal, mixed-methods, and community-engaged designs that can capture collective resilience processes without reducing them to individual-level variables. In the context of ongoing conflicts, displacement, and humanitarian crises affecting the Middle East and Gulf region, sumud warrants renewed scholarly and clinical attention as a culturally grounded framework for resilience-informed psychotherapeutic and psychosocial practice.
