# Supplementary material for: Sumud as a framework for collective resilience: a systematic review of implications for transcultural psychiatry and psychotherapy
Source: Front Psychiatry. 2026 Oct 16;17:1908428. doi: 10.3389/fpsyt.2026.1908428 (PMC13721743; doi:10.3389/fpsyt.2026.1908428)
Supplement: Supplementary file 1 [file Table1.docx]

**Supplementary Table 1. PRISMA 2020 Checklist**

**Reported: Yes** (green) **Partial** (amber) **N/A** (grey — not applicable to narrative review without meta-analysis)

| **Section** | **#** | **Checklist Item** | **Reported?** | **Location in Manuscript** | **Page(s)** |
| --- | --- | --- | --- | --- | --- |
| **TITLE & ABSTRACT** | | | | | |
| **Title / Abstract** | **1** | Identify the report as a systematic review in the title. | **Yes** | *Title page* | 1 |
| **Title / Abstract** | **2** | Provide a structured summary including, as applicable: background; objectives; eligibility criteria; information sources; risk of bias; synthesis methods; results; limitations; conclusions and implications of key findings; funding; registration. | **Yes** | *Abstract — Background, Aims, Method, Results, Conclusions* | 2-3 |
| **INTRODUCTION** | | | | | |
| **Introduction** | **3** | Describe the rationale for the review in the context of existing knowledge. | **Yes** | *Introduction — paragraphs 1–5* | 4-7 |
| **Introduction** | **4** | Provide an explicit statement of the objectives or questions the review addresses. | **Yes** | *Introduction — final paragraph (aims to synthesise sumud conceptualisations and examine psychotherapy implications)* | 7 |
| **METHODS** | | | | | |
| **Methods** | **5** | Specify the inclusion and exclusion criteria for the review and how studies were grouped for the syntheses. | **Yes** | *Method: Eligibility criteria — inclusion (3 criteria) and exclusion (4 criteria)* | 9–10 |
| **Methods** | **6** | Specify all databases, registers, websites, handsearching, organisations, reference lists and other sources searched or consulted to identify studies. Specify the date when each source was last searched or consulted. | **Yes** | *Method: Data sources and search strategy — PubMed, Scopus, WoS, Embase; searched February–March 2026 (covering all publications to search date); manual searching of reference lists; rapid update search conducted 1 June 2026* | 7-9 |
| **Methods** | **7** | Present the full search strategies for all databases, registers, and websites, including any filters and limits used. | **Yes** | *Method: Data sources — representative search string; full strategies in Supplementary Table 1* | 9 |
| **Methods** | **8** | Specify the methods used to decide whether a study met the inclusion criteria of the review, including how many reviewers screened each record and each report retrieved, whether they worked independently, and if applicable, details of automation tools used in the process. | **Yes** | *Method: Study selection — dual independent T&A screening (D.A.E. & N.T.D.); dual independent full-text screening (D.A.E. & N.T.D.); conflicts resolved by discussion; third reviewer (I.E.) consulted where consensus not reached; final decision by majority agreement* | 10–11 |
| **Methods** | **9** | Specify the methods used to collect data from reports, including how many reviewers collected data from each report, whether they worked independently, any processes for obtaining or confirming data from study investigators, and if applicable, details of automation tools used in the process. | **Yes** | *Method: Data extraction — dual independent by D.A.E. & N.T.D.; 20% sample independently verified by I.E.; conflicts resolved by discussion, adjudication by I.E. where needed; extraction commenced March 2026, finalized 30 April 2026; update search extraction completed June 2026* | 11–12 |
| **Methods** | **10a** | List and define all outcomes for which data were sought. Specify whether all results that were compatible with each outcome domain in each study were sought, and if not, the methods used to prioritise result selection. | **N/A** | *Not applicable — no quantitative outcomes sought; review is narrative/conceptual* | — |
| **Methods** | **10b** | List and define all other variables for which data were sought (e.g. participant and intervention characteristics, funding sources). Describe any assumptions made about any missing or unclear information. | **Yes** | *Method: Data extraction — extraction matrix items (1)–(7): publication characteristics, study design, population, sumud conceptualisation, resilience dimensions, cultural elements, MH/psychotherapy implications* | 12 |
| **Methods** | **11a** | Describe the methods used to assess risk of bias in the included studies, including details of the tool(s) used, how many reviewers assessed each study and whether they worked independently, and if applicable, details of automation tools used in the process. | **Yes** | *Method: Quality appraisal — narrative appraisal via analytical matrix; formal RoB tools not applied (rationale stated); attention to methodological clarity, conceptual coherence, sumud definition explicitness* | 12–13 |
| **Methods** | **11b** | Describe any methods used to assess risk of bias that might affect the cumulative evidence (e.g. publication bias, selective reporting bias). | **Partial** | *Method: Quality appraisal and Discussion: Methodological strengths and gaps — geographic concentration and qualitative dominance noted as potential sources of bias in evidence base* | 12–13, 23 |
| **Methods** | **12** | Describe the methods used to present and summarise the findings. If meta-analysis was done, describe the models, method to identify the presence and extent of statistical heterogeneity, and software package used. | **Yes** | *Method: Data extraction and analytical approach — iterative inductive thematic synthesis using extraction matrix; no meta-analysis (narrative systematic review)* | 11–12 |
| **Methods** | **13** | Describe planned methods for investigating causes of heterogeneity among study results (e.g. subgroup analysis, meta-regression). If not done, state this. | **N/A** | *Not applicable — no quantitative synthesis; heterogeneity addressed narratively through subgroup discussion (gender, developmental, educational dimensions)* | — |
| **Methods** | **14** | Describe any sensitivity analyses conducted to assess robustness of the synthesised results. | **N/A** | *Not applicable — narrative synthesis; no meta-analysis performed* | — |
| **RESULTS** | | | | | |
| **Results** | **15** | Describe the results of the search and selection process, from the number of records identified in the search to the number of studies included in the review, ideally using a flow diagram. | **Yes** | *Method: Study selection; Results: Overview; Fig. 1 (PRISMA 2020 flow diagram) — 178 records identified → 111 screened → 37 included from original search; 2 additional studies identified through June 2026 update search → 39 total included* | 10–11, 13 |
| **Results** | **16a** | Cite each included study and present its key characteristics. | **Yes** | *Results: Overview of included studies; Table 1 — all 39 studies with design, population, sumud conceptualisation, resilience dimensions, MH relevance, discipline (2 studies added from update search marked with asterisk)* | 13, 36–39 |
| **Results** | **16b** | Cite each excluded study that might be expected to be eligible but was excluded, and explain why it was excluded. | **Partial** | *Method: Study selection — reasons for full-text exclusion described categorically; individual excluded studies not listed (consistent with narrative review convention; available on request from corresponding author)* | 10–11 |
| **Results** | **17** | For each included study, present the assessed risk of bias. | **Yes** | *Method: Quality appraisal — narrative appraisal embedded in analytical matrix; Results: Methodological patterns and gaps — patterns across studies discussed* | 12–13, 17–18 |
| **Results** | **18** | For all outcomes, present, for each study: (a) summary statistics for each group; (b) an effect estimate and its precision; and, if using meta-analysis, the pooled estimate and its precision. | **N/A** | *Not applicable — no quantitative synthesis or meta-analysis; findings presented as thematic narrative summary* | — |
| **Results** | **19** | Describe all statistical analyses, heterogeneity measures, sensitivity analyses, and subgroup analyses. | **N/A** | *Not applicable — narrative systematic review; no meta-analysis* | — |
| **Results** | **20** | Present results of any assessment of publication bias, small-study bias, and certainty of evidence. | **N/A** | *Not applicable — narrative synthesis; no meta-analysis* | — |
| **Results** | **21** | Present results of all planned sensitivity analyses. | **N/A** | *Not applicable — narrative synthesis* | — |
| **DISCUSSION** | | | | | |
| **Discussion** | **22** | Provide a general interpretation of the results in the context of other evidence. | **Yes** | *Discussion — Reconceptualising resilience; Embodied/cultural expressions; Developmental and gendered dimensions; Implications for psychotherapy practice (pp. 19–23)* | 25-29 |
| **Discussion** | **23** | Discuss any limitations of the evidence included in the review. | **Yes** | *Discussion: Methodological strengths and gaps; Contributions and limitations — qualitative dominance, geographic concentration, absence of intervention studies, operationalisation challenges* | 25–34 |
| **Discussion** | **24** | Describe any implications of the results for practice, policy, or future research. | **Yes** | *Discussion: Implications for psychotherapy practice; Conclusion — psychotherapy modalities, staff support, service design, future mixed-methods research* | 25–34 |
| **OTHER INFORMATION** | | | | | |
| **Other** | **25** | Provide information about the funding for the review and the role of the funders in the review. | **Yes** | *Declarations: Funding Statement — no funding received* | 35 |
| **Other** | **26** | Provide the name and registration information for the protocol, including the name and URL of the registration database, the unique ID number, and the version and date of the most recent protocol. | **Yes** | *Method: Review design; Declarations: PROSPERO Registration — CRD420261279052, registered 5 Feb 2026* | 8, 36 |
| **Other** | **27** | Indicate where the completed PRISMA checklist can be accessed. | **Yes** | *Submitted as a separate document accompanying the submission to Frontiers in Psychiatry (Supplementary Table 1 — Sumud_PRISMA2020_Checklist_June2026.docx)* | — |

*Note: Items rated N/A are not applicable to this systematic narrative review without meta-analysis, in accordance with PRISMA 2020 guidance (Page et al., BMJ, 2021). Items rated Partial are reported in principle but with acknowledged limitations noted in the manuscript. Page numbers refer to the manuscript version submitted to Frontiers in Psychiatry, June 2026.*
