# Supplementary material for: Sumud as a framework for collective resilience: a systematic review of implications for transcultural psychiatry and psychotherapy
Source: Front Psychiatry. 2026 Oct 16;17:1908428. doi: 10.3389/fpsyt.2026.1908428 (PMC13721743; doi:10.3389/fpsyt.2026.1908428)
Supplement: Supplementary file 2 [file Table2.docx]

**Supplementary Table 2. Search Strings**

| **Database** | **String** | **Results** | **Last search** |
| --- | --- | --- | --- |
| PubMed- Cultural | **(("sumud"[Title/Abstract] OR "sumoud"[Title/Abstract] OR "samoud"[Title/Abstract] OR "somoud"[Title/Abstract]) AND ("steadfastness"[Title/Abstract] OR "resilien*"[Title/Abstract] OR "psychological resilien*"[Title/Abstract] OR "coping"[Title/Abstract] OR "coping strateg*"[Title/Abstract] OR "endurance"[Title/Abstract] OR "adaptation"[Title/Abstract] OR "resilience, psychological"[MeSH Terms] OR "resilience, psychological"[MeSH Terms] OR "stress, psychological"[MeSH Terms] OR "adaptation, psychological"[MeSH Terms]) AND ("culture*"[Title/Abstract] OR "cultural practice*"[Title/Abstract] OR "collective identity"[Title/Abstract] OR "faith"[Title/Abstract] OR "religion"[Title/Abstract] OR "spirituality"[Title/Abstract] OR "art"[Title/Abstract] OR "poetry"[Title/Abstract] OR "theatre*"[Title/Abstract] OR "cinema"[Title/Abstract] OR "dabke*"[Title/Abstract]))** Filters: **English** | [6](https://pubmed.ncbi.nlm.nih.gov/?term=%28%28%22sumud%22%5BTitle%2FAbstract%5D+OR+%22sumoud%22%5BTitle%2FAbstract%5D+OR+%22samoud%22%5BTitle%2FAbstract%5D+OR+%22somoud%22%5BTitle%2FAbstract%5D%29+AND+%28%22steadfastness%22%5BTitle%2FAbstract%5D+OR+%22resilien%2A%22%5BTitle%2FAbstract%5D+OR+%22psychological+resilien%2A%22%5BTitle%2FAbstract%5D+OR+%22coping%22%5BTitle%2FAbstract%5D+OR+%22coping+strateg%2A%22%5BTitle%2FAbstract%5D+OR+%22endurance%22%5BTitle%2FAbstract%5D+OR+%22adaptation%22%5BTitle%2FAbstract%5D+OR+%22resilience%2C+psychological%22%5BMeSH+Terms%5D+OR+%22resilience%2C+psychological%22%5BMeSH+Terms%5D+OR+%22stress%2C+psychological%22%5BMeSH+Terms%5D+OR+%22adaptation%2C+psychological%22%5BMeSH+Terms%5D%29+AND+%28%22culture%2A%22%5BTitle%2FAbstract%5D+OR+%22cultural+practice%2A%22%5BTitle%2FAbstract%5D+OR+%22collective+identity%22%5BTitle%2FAbstract%5D+OR+%22faith%22%5BTitle%2FAbstract%5D+OR+%22religion%22%5BTitle%2FAbstract%5D+OR+%22spirituality%22%5BTitle%2FAbstract%5D+OR+%22art%22%5BTitle%2FAbstract%5D+OR+%22poetry%22%5BTitle%2FAbstract%5D+OR+%22theatre%22%5BTitle%2FAbstract%5D+OR+%22cinema%22%5BTitle%2FAbstract%5D+OR+%22dabke%22%5BTitle%2FAbstract%5D%29%29&filter=lang.english&size=200&sort=relevance) | 29-12-2025 |
| PubMed- Psychological | **(((((((sumud[Title/Abstract]) ) OR (sumoud[Title/Abstract])) OR (samoud[Title/Abstract])) OR (somoud[Title/Abstract]))) AND ((((((((((((steadfastness[Title/Abstract]) OR (resilien*[Title/Abstract])) OR ("psychological resilien*"[Title/Abstract])) OR (coping[Title/Abstract])) OR ("coping strateg*"[Title/Abstract])) OR (endurance[Title/Abstract])) OR (adaptation[Title/Abstract])) OR (resilience[MeSH Terms])) OR (Resilience, Psychological[MeSH Terms])))) OR (Stress, Psychological[MeSH Terms]) OR (Adaptation, Psychological[MeSH Terms]))) AND (((((((((psychotherap*[Title/Abstract]) OR (counseling[Title/Abstract])) OR (therap*[Title/Abstract])) OR ("mental health"[Title/Abstract])) OR ("psychological practice"[Title/Abstract]))) OR (Psychotherapy[MeSH Terms])) OR (Counseling[MeSH Terms])) OR (mental health services[MeSH Terms])) Filters: English** | [8](https://pubmed.ncbi.nlm.nih.gov/?term=%28%28%28%28%28%28%28sumud%5BTitle%2FAbstract%5D%29+%29+OR+%28sumoud%5BTitle%2FAbstract%5D%29%29+OR+%28samoud%5BTitle%2FAbstract%5D%29%29+OR+%28somoud%5BTitle%2FAbstract%5D%29%29%29+AND+%28%28%28%28%28%28%28%28%28%28%28%28steadfastness%5BTitle%2FAbstract%5D%29+OR+%28resilien%2A%5BTitle%2FAbstract%5D%29%29+OR+%28%22psychological+resilien%2A%22%5BTitle%2FAbstract%5D%29%29+OR+%28coping%5BTitle%2FAbstract%5D%29%29+OR+%28%22coping+strateg%2A%22%5BTitle%2FAbstract%5D%29%29+OR+%28endurance%5BTitle%2FAbstract%5D%29%29+OR+%28adaptation%5BTitle%2FAbstract%5D%29%29+OR+%28resilience%5BMeSH+Terms%5D%29%29+OR+%28Resilience%2C+Psychological%5BMeSH+Terms%5D%29%29%29%29+OR+%28Stress%2C+Psychological%5BMeSH+Terms%5D%29+OR+%28Adaptation%2C+Psychological%5BMeSH+Terms%5D%29%29%29+AND+%28%28%28%28%28%28%28%28%28psychotherap%2A%5BTitle%2FAbstract%5D%29+OR+%28counseling%5BTitle%2FAbstract%5D%29%29+OR+%28therap%2A%5BTitle%2FAbstract%5D%29%29+OR+%28%22mental+health%22%5BTitle%2FAbstract%5D%29%29+OR+%28%22psychological+practice%22%5BTitle%2FAbstract%5D%29%29%29+OR+%28Psychotherapy%5BMeSH+Terms%5D%29%29+OR+%28Counseling%5BMeSH+Terms%5D%29%29+OR+%28mental+health+services%5BMeSH+Terms%5D%29%29&filter=lang.english&size=200&sort=relevance) |  |
|  |  |  |  |
| Scopus- Cultural | ( ( TITLE-ABS-KEY ( sumud ) OR TITLE-ABS-KEY ( sumoud ) OR TITLE-ABS-KEY ( samoud ) OR TITLE-ABS-KEY ( somoud ) ) ) AND ( ( TITLE-ABS-KEY ( steadfastness ) OR TITLE-ABS-KEY ( resilien* ) OR TITLE-ABS-KEY ( "psychological resilien*" ) OR TITLE-ABS-KEY ( coping ) OR TITLE-ABS-KEY ( "coping strateg*" ) OR TITLE-ABS-KEY ( endurance ) OR TITLE-ABS-KEY ( adaptation ) OR TITLE-ABS-KEY ( "Resilience, Psychological" ) OR TITLE-ABS-KEY ( "Psychological Resilienc*" ) OR TITLE-ABS-KEY ( "Resilient Response*, Psychological" ) OR TITLE-ABS-KEY ( "Psychological Resilient Response*" ) OR TITLE-ABS-KEY ( "Stress Immunit*" ) OR TITLE-ABS-KEY ( "Psychologic* Stress*" ) OR TITLE-ABS-KEY ( "Stress*, Psychologic*" ) OR TITLE-ABS-KEY ( "Life Stress*" ) OR TITLE-ABS-KEY ( "Emotional Stress" ) OR TITLE-ABS-KEY ( "Psychologic* Stressful Condition*" ) OR TITLE-ABS-KEY ( "Condition*, Psychologically" ) OR TITLE-ABS-KEY ( "Psychological Stress Experience*" ) OR TITLE-ABS-KEY ( "Individual Stressor*" ) OR TITLE-ABS-KEY ( "Psychological Cumulative Stress*" ) OR TITLE-ABS-KEY ( "Psychological Stress Overload*" ) OR TITLE-ABS-KEY ( "Psychological Stress Measurement*" ) OR TITLE-ABS-KEY ( "Psychological Stress Processe*" ) OR TITLE-ABS-KEY ( "Adaptation, Psychological" ) OR TITLE-ABS-KEY ( "Psychologic* Adaptation" ) OR TITLE-ABS-KEY ( "Adaptive Behavior*" ) OR TITLE-ABS-KEY ( Adjustment ) OR TITLE-ABS-KEY ( "Healthy Adaptation*" ) OR TITLE-ABS-KEY ( "Positive Adaptation*" ) OR TITLE-ABS-KEY ( "Psychological Recover*" ) ) ) AND ( ( TITLE-ABS-KEY ( cultur* ) OR TITLE-ABS-KEY ( "cultural practice*" ) OR TITLE-ABS-KEY ( "collective identity" ) OR TITLE-ABS-KEY ( faith ) OR TITLE-ABS-KEY ( religion ) OR TITLE-ABS-KEY ( spirituality ) OR TITLE-ABS-KEY ( art ) OR TITLE-ABS-KEY ( poetry ) OR TITLE-ABS-KEY ( theatre* ) OR TITLE-ABS-KEY ( cinema ) OR TITLE-ABS-KEY ( dabke* ) ) ) AND ( LIMIT-TO ( LANGUAGE , "English" ) ) | 30 | 30-12-2025 |
| Scopus- Psychological | ( ( TITLE-ABS-KEY ( sumud ) OR TITLE-ABS-KEY ( sumoud ) OR TITLE-ABS-KEY ( samoud ) OR TITLE-ABS-KEY ( somoud ) ) ) AND ( ( TITLE-ABS-KEY ( steadfastness ) OR TITLE-ABS-KEY ( resilien* ) OR TITLE-ABS-KEY ( "psychological resilien*" ) OR TITLE-ABS-KEY ( coping ) OR TITLE-ABS-KEY ( "coping strateg*" ) OR TITLE-ABS-KEY ( endurance ) OR TITLE-ABS-KEY ( adaptation ) OR TITLE-ABS-KEY ( "Resilience, Psychological" ) OR TITLE-ABS-KEY ( "Psychological Resilienc*" ) OR TITLE-ABS-KEY ( "Resilient Response*, Psychological" ) OR TITLE-ABS-KEY ( "Psychological Resilient Response*" ) OR TITLE-ABS-KEY ( "Stress Immunit*" ) OR TITLE-ABS-KEY ( "Psychologic* Stress*" ) OR TITLE-ABS-KEY ( "Stress*, Psychologic*" ) OR TITLE-ABS-KEY ( "Life Stress*" ) OR TITLE-ABS-KEY ( "Emotional Stress" ) OR TITLE-ABS-KEY ( "Psychologic* Stressful Condition*" ) OR TITLE-ABS-KEY ( "Condition*, Psychologically" ) OR TITLE-ABS-KEY ( "Psychological Stress Experience*" ) OR TITLE-ABS-KEY ( "Individual Stressor*" ) OR TITLE-ABS-KEY ( "Psychological Cumulative Stress*" ) OR TITLE-ABS-KEY ( "Psychological Stress Overload*" ) OR TITLE-ABS-KEY ( "Psychological Stress Measurement*" ) OR TITLE-ABS-KEY ( "Psychological Stress Processe*" ) OR TITLE-ABS-KEY ( "Adaptation, Psychological" ) OR TITLE-ABS-KEY ( "Psychologic* Adaptation" ) OR TITLE-ABS-KEY ( "Adaptive Behavior*" ) OR TITLE-ABS-KEY ( Adjustment ) OR TITLE-ABS-KEY ( "Healthy Adaptation*" ) OR TITLE-ABS-KEY ( "Positive Adaptation*" ) OR TITLE-ABS-KEY ( "Psychological Recover*" ) ) ) AND ( ( TITLE-ABS-KEY ( psychotherap* ) OR TITLE-ABS-KEY ( counseling ) OR TITLE-ABS-KEY ( therap* ) OR TITLE-ABS-KEY ( "mental health" ) OR TITLE-ABS-KEY ( "mental health service*" ) OR TITLE-ABS-KEY ( "Health Service*, Mental" ) OR TITLE-ABS-KEY ( "Mental Health Service*" ) OR TITLE-ABS-KEY ( "Hygiene Service*, Mental" ) OR TITLE-ABS-KEY ( "Mental Hygiene Service*" ) ) ) AND ( LIMIT-TO ( LANGUAGE , "English" ) ) | 10 | 30-12-2025 |
|  |  |  |  |
| Embase- Cultural | (**'sumud'**:ti,ab,kw OR **'sumoud'**:ti,ab,kw OR **'samoud'**:ti,ab,kw OR **'somoud'**:ti,ab,kw) AND (**'steadfastness'**:ti,ab,kw OR **'resilien*'**:ti,ab,kw OR **'psychological resilien*'**:ti,ab,kw OR **'coping'**:ti,ab,kw OR **'coping strateg*'**:ti,ab,kw OR **'endurance'**:ti,ab,kw OR **'adaptation'**:ti,ab,kw OR **'psychological resilience'**/exp OR **'mental stress'**/exp OR **'psychological adjustment'**/exp) AND (**'culture*'**:ti,ab,kw OR **'cultural practice*'**:ti,ab,kw OR **'collective identity'**:ti,ab,kw OR **'faith'**:ti,ab,kw OR **'religion'**:ti,ab,kw OR **'spirituality'**:ti,ab,kw OR **'art'**:ti,ab,kw OR **'poetry'**:ti,ab,kw OR **'theatre'**:ti,ab,kw OR **'cinema'**:ti,ab,kw OR **'dabke'**:ti,ab,kw) AND [english]/lim | **5** | 29-12-2025 |
| Embase- Psychological | (**'sumud'**:ti,ab,kw OR **'sumoud'**:ti,ab,kw OR **'samoud'**:ti,ab,kw OR **'somoud'**:ti,ab,kw) AND (**'steadfastness'**:ti,ab,kw OR **'resilien*'**:ti,ab,kw OR **'psychological resilien*'**:ti,ab,kw OR **'coping'**:ti,ab,kw OR **'coping strateg*'**:ti,ab,kw OR **'endurance'**:ti,ab,kw OR **'adaptation'**:ti,ab,kw OR **'resilience'**/exp OR **'psychological resilience'**/exp OR **'mental stress'**/exp OR **'psychological adjustment'**/exp) AND (**'psychotherap*'**:ti,ab,kw OR **'counseling'**:ti,ab,kw OR **'therap*'**:ti,ab,kw OR **'mental health'**:ti,ab,kw OR **'psychological practice'**:ti,ab,kw OR **'psychotherapy'**/exp OR **'counseling'**/exp OR **'mental health service'**/exp) AND [english]/lim | **8** |  |
|  |  |  |  |
| WOS- Cultural | **(((TS=(sumud)) OR TS=( sumoud )) OR TS=(samoud )) OR TS=(somoud ) AND ((((((((((((((((((((((((((((((TS=(steadfastness )) OR TS=(resilien* )) OR TS=( "psychological resilien*" )) OR TS=( coping)) OR TS=( "coping strateg*" )) OR TS=( endurance )) OR TS=( adaptation)) OR TS=("Resilience, Psychological" )) OR TS=("Psychological Resilienc*" )) OR TS=("Resilient Response*, Psychological")) OR TS=("Psychological Resilient Response*" )) OR TS=( "Stress Immunit*" )) OR TS=("Psychologic* Stress*" )) OR TS=( "Stress*, Psychologic*" )) OR TS=("Life Stress*")) OR TS=("Emotional Stress" )) OR TS=("Psychologic* Stressful Condition*" )) OR TS=("Condition*, Psychologically")) OR TS=("Psychological Stress Experience*" )) OR TS=("Individual Stressor*" )) OR TS=("Psychological Cumulative Stress*" )) OR TS=("Psychological Stress Overload*" )) OR TS=( "Psychological Stress Measurement*" )) OR TS=("Psychological Stress Processe*" )) OR TS=("Adaptation, Psychological")) OR TS=("Psychologic* Adaptation" )) OR TS=( "Adaptive Behavior*" )) OR TS=(Adjustment )) OR TS=("Healthy Adaptation*" )) OR TS=( "Positive Adaptation*" )) OR TS=("Psychological Recover*" ) AND ((((((((((TS=(cultur* )) OR TS=("cultural practice*" )) OR TS=("collective identity" )) OR TS=(faith)) OR TS=(religion)) OR TS=(spirituality)) OR TS=( art )) OR TS=(poetry )) OR TS=(theatre* )) OR TS=( cinema )) OR TS=(dabke*) and English (Languages)** | [23](https://www-webofscience-com.uaeu.idm.oclc.org/wos/woscc/summary/f0657040-7a92-4dc2-938b-1ddc6c47a0e9-0194a2fa49/relevance/1) |  |
| WOS- Psychological | **(((TS=(sumud)) OR TS=( sumoud )) OR TS=(samoud )) OR TS=(somoud ) AND ((((((((((((((((((((((((((((((TS=(steadfastness )) OR TS=(resilien* )) OR TS=( "psychological resilien*" )) OR TS=( coping)) OR TS=( "coping strateg*" )) OR TS=( endurance )) OR TS=( adaptation)) OR TS=("Resilience, Psychological" )) OR TS=("Psychological Resilienc*" )) OR TS=("Resilient Response*, Psychological")) OR TS=("Psychological Resilient Response*" )) OR TS=( "Stress Immunit*" )) OR TS=("Psychologic* Stress*" )) OR TS=( "Stress*, Psychologic*" )) OR TS=("Life Stress*")) OR TS=("Emotional Stress" )) OR TS=("Psychologic* Stressful Condition*" )) OR TS=("Condition*, Psychologically")) OR TS=("Psychological Stress Experience*" )) OR TS=("Individual Stressor*" )) OR TS=("Psychological Cumulative Stress*" )) OR TS=("Psychological Stress Overload*" )) OR TS=( "Psychological Stress Measurement*" )) OR TS=("Psychological Stress Processe*" )) OR TS=("Adaptation, Psychological")) OR TS=("Psychologic* Adaptation" )) OR TS=( "Adaptive Behavior*" )) OR TS=(Adjustment )) OR TS=("Healthy Adaptation*" )) OR TS=( "Positive Adaptation*" )) OR TS=("Psychological Recover*" ) AND ((((((((TS=(psychotherap* )) OR TS=( counseling )) OR TS=( therap* )) OR TS=("mental health")) OR TS=("mental health service*")) OR TS=("Health Service*, Mental" )) OR TS=( "Mental Health Service*" )) OR TS=( "Hygiene Service*, Mental")) OR TS=("Mental Hygiene Service*") and English (Languages)** | [12](https://www-webofscience-com.uaeu.idm.oclc.org/wos/woscc/summary/3dca41b0-098d-4567-ae37-c724594457b8-0194a30f20/relevance/1) |  |
|  |  |  |  |
