# Supplementary material for: Sumud as a framework for collective resilience: a systematic review of implications for transcultural psychiatry and psychotherapy
Source: Front Psychiatry. 2026 Oct 16;17:1908428. doi: 10.3389/fpsyt.2026.1908428 (PMC13721743; doi:10.3389/fpsyt.2026.1908428)
Supplement: Supplementary file 3 [file Table3.docx]

**Supplementary Table 3. Data Extraction Matrix**

*Sumud as Collective Resilience: A Systematic Review of Cultural, Relational, and Psychotherapeutic Implications| n = 39 included studies*

| PROSPERO: CRD420261279052

| **Study (Author, Year)** | **Context / Setting** | **Study Design** | **Population** | **Conceptualisation of Sumud** | **Key Resilience Dimensions** | **Mental Health / Psychotherapy Relevance** | **Discipline** |
| --- | --- | --- | --- | --- | --- | --- | --- |
| 1. **Abudayya (2025)** | Gaza Strip, occupied Palestinian territory | Qualitative — in-depth interviews | Civilians under blockade and military operations | Everyday coping and endurance under siege conditions; sumud as normalisation of adversity and maintenance of daily function amid extreme constraint | Psychological: meaning-making, emotional containment; Social: community solidarity, mutual aid; Cultural: continuation of domestic and civic routine as resistance | Supportive and trauma-informed approaches; importance of community-based psychological first aid; cautions against pathologising survival behaviour; affirms dignity-centred care | Psychology / Global Mental Health |
| 2. **Abuelaish & Yousufzai (2023)** | Contexts of protracted conflict and siege (conceptual; Palestinian and international) | Conceptual / theoretical paper | Conflict-affected civilian populations (general) | Sumud as a paradigm shift in resilience thinking: moves beyond 'bouncing back' to sustained moral endurance without resolution; proposes sumud as a new conceptual lens for global mental health | Psychological: hope, dignity, purpose; Moral: ethical commitment to life under siege; Collective: communal bonds as sustaining infrastructure; Institutional: health systems as sites of sumud | Argues for community-centred mental health models; critiques biomedical deficit frameworks; supports integration of sumud into global MH policy and service design | Psychiatry / Global Mental Health |
| 3. **Abushalha (2022)** | Palestinian theatrical arts; divided Palestinian territories | Performance analysis / cultural critique | Theatre artists, performers, and audiences (Palestinian) | Sumud enacted through theatrical production as a form of symbolic unity and national identity affirmation; art as the vehicle through which fragmented communities sustain shared narrative | Cultural: artistic expression as collective identity anchor; Symbolic: theatre as ritual of belonging; Relational: shared audience experience builds communal bonds | Relevance for arts-based and drama therapy approaches; demonstrates how performance creates witnessing communities; supports use of theatre in group psychosocial work | Humanities / Performance Studies |
| 4. **Al Jayousi & Nishide (2025)** | Palestinian NGO sector | Qualitative — interviews with NGO staff | Staff and managers of Palestinian civil society organisations | Organisational sumud: resilience sustained through knowledge-sharing, institutional memory, and collective professional identity within civil society; organisations as repositories of communal endurance | Institutional: organisational culture and shared purpose; Relational: collegial networks, professional solidarity; Cognitive: knowledge management as resilience infrastructure | Informs organisational mental health and staff support; peer consultation and knowledge-sharing circles as resilience strategies; relevant to supervision models in humanitarian settings | Development Studies / Organisational Psychology |
| 5. **Aqtam et al. (2025)** | Conflict-affected Palestinian communities (systematic review scope) | Systematic review of mental health interventions | Conflict-affected Palestinian populations (all ages) | Sumud embedded within a broader review of resilience-informed mental health interventions; collective endurance as framework for culturally grounded care in conflict settings | Multilevel: individual coping, community-based social support, institutional health infrastructure; Cultural identity as protective factor across levels | Advocates for culturally grounded and community-oriented mental health interventions; critiques individually focused CBT-dominant models as insufficient in collective adversity contexts | Psychiatry / Global Mental Health |
| 6. **Atallah (2017)** | Palestinian refugee communities (camp settings; Jordan and Lebanon) | Community-based qualitative; participatory action research | Palestinian refugee families across generations | Intergenerational collective resilience: sumud transmitted through shared narratives, oral history, cultural practices, and familial memory; community as the primary resilience unit; structural violence as context shaping endurance | Collective: community as resilience unit; Intergenerational: narrative transmission of memory and values; Cultural: oral history, shared practices; Moral: collective responsibility to sustain identity | Directly informs family-based and narrative therapy; community-level interventions that engage family storytelling; demonstrates value of culturally embedded group work; cautions against individually focused trauma therapy in collective adversity | Psychology / Transcultural Psychiatry |
| 7. **Awayed-Bishara (2025)** | Palestinian schools in Israel (formal education system) | Linguistic ethnography | Palestinian youth and teachers in Israeli state schools | Sumud pedagogy as linguistic citizenship: teachers and students enact sumud through language practices, resisting imposed subjectivities and sustaining Palestinian identity through Arabic language use and narrative in formal schooling contexts | Cultural: language as identity anchor; Educational: pedagogy as site of collective resistance; Relational: teacher–student alliances in resisting assimilation; Individual: self-affirmation through linguistic agency | Relevant to school-based mental health and psychoeducational programmes; supports narrative and identity-affirming approaches in educational settings; demonstrates how language can be a therapeutic and resilience-building tool | Sociolinguistics / Education |
| 8. **Ayesh & Ben Hagai (2025)** | Occupied Palestinian territory (West Bank and Gaza) | Qualitative — interview study | Palestinian adults (community sample) | Sumud as everyday resistance and psychological recovery: endurance not as passivity but as active relational awareness, contextual appraisal, and moral stance within conditions of prolonged occupation | Psychological: appraisal, awareness, agency; Relational: social connection and mutual recognition; Moral: ethical positioning as component of recovery | Supports context-aware psychotherapy that validates political and structural realities; cautions against decontextualised trauma models; affirms relational and existential dimensions of psychological recovery | Psychology / Health Psychology |
| 9. **Bascunan-Wiley (2019)** | Palestinian diaspora community in Santiago, Chile | Ethnography / food studies | Palestinian-Chilean families (diaspora; multi-generational) | Culinary sumud: preparation, sharing, and transmission of Palestinian food practices as a form of collective memory, place attachment, and diasporic identity maintenance; cuisine as intergenerational resilience ritual | Cultural: food as memory and identity; Intergenerational: recipe transmission across generations; Relational: communal meals as bonding ritual; Embodied: sensory connection to homeland | Highlights role of everyday ritual and embodied cultural practices in sustaining wellbeing in diaspora; supports culturally responsive approaches that incorporate food, ritual, and memory; relevant to narrative and meaning-centred therapy | Anthropology / Diaspora Studies |
| 10. **Bosqui et al. (2020)** | Lebanon (community settings; conflict-affected adolescents) | Pilot psychoeducational intervention | Adolescents facing adversity in Lebanon | 'Somoud' (Arabic: steadfastness) operationalised as a psychoeducational concept in a comic book format to convey evidence-based mental health and coping messages; translates collective resilience language into accessible youth-oriented resources | Psychological: coping skills, emotional literacy; Social: peer connection and social support; Cultural: use of culturally resonant language (somoud) to frame resilience as familiar and achievable | Directly relevant: demonstrates feasibility of adapting sumud concept into structured youth mental health tools; supports psychoeducational and creative approaches for adolescents; informs preventive MH programming in conflict-affected settings | Psychology / Global Mental Health |
| 11. **Busse (2022)** | Occupied Palestinian territory (everyday urban and rural life) | Spatial analysis / political geography | Palestinian civilians (community and household level) | Sumud as quotidian spatial practice: endurance enacted through everyday activities — cooking, gardening, movement, domestic maintenance — that assert presence and continuity of ordinary life in contested space | Embodied: bodily engagement with place; Spatial: occupation of contested territory as act of endurance; Cultural: everyday routines as expressions of collective belonging; Temporal: persistence of ordinary life across time | Cautions against pathologising ordinary life under occupation; affirms dignity in the mundane; supports therapeutic approaches that recognise value of everyday routine and place-based identity in treatment planning | Political Geography / Social Science |
| 12. **Dabbagh et al. (2023)** | Palestinian territories (national health policy context) | Policy strategy paper / system-level analysis | Children and adolescents (population-level; Palestinian national context) | Sumud embedded in national CAMH strategy: collective and community resilience as foundational principle for child and adolescent mental health service design; institutional sumud — sustaining services under occupation and resource constraint | Institutional: health system design; Collective: community as protective environment; Developmental: child- and family-centred frameworks; Cultural: sumud as value orienting service priorities | Directly informs CAMH service design; supports family-centred, community-based, and prevention-oriented mental health systems; demonstrates how sumud can frame national MH strategy; relevant to policy-level clinical leadership | Psychiatry / Child & Adolescent Mental Health |
| 13. **Elkhalid et al. (2025)** | Palestinian families (diaspora and homeland settings) | Narrative interviews / communication analysis | Palestinian parents and children across generations | Intergenerational storytelling as sumud: families sustain identity, connection to place (Palestine), and cultural values through deliberate narrative practices across generations; stories as vehicles of memory, grief, and continuity | Intergenerational: narrative transmission; Relational: parent–child bonds through shared storytelling; Cultural: oral history as identity maintenance; Psychological: meaning-making through narrative | Strongly supports narrative family therapy and genogram-based approaches; demonstrates clinical value of eliciting family stories, displacement narratives, and intergenerational memory in therapeutic work; relevant to diaspora and refugee mental health practice | Communication Studies / Family Studies |
| 14. **El-Shakry (2021)** | Palestinian cultural and literary production (theoretical) | Conceptual / postcolonial literary analysis | — (conceptual; no specific study population) | Sumud as existential orientation toward a 'future impossible': endurance not grounded in optimism but in ethical commitment to a future that cannot be guaranteed; aesthetic and existential dimensions of Palestinian cultural production as sumud | Existential: hope without certainty; Aesthetic: art as existential stance; Ethical: commitment to possibility under foreclosure; Cultural: literary and artistic work as collective meaning-making | Deepens existential and meaning-centred perspectives in psychotherapy; relevant to Frankl's logotherapy and Yalom's existential psychiatry; supports dignity-centred formulation; cautions against false hope in therapeutic work with traumatised communities | Postcolonial Studies / Humanities |
| 15. **Groten (2025)** | Gaza coastal communities (fishing villages) | Ethnography | Palestinian fishermen and fishing community members | Fishing as sumud: livelihood practice under military restriction reframed as endurance and attachment to place and occupation; risk-taking and persistence in the face of maritime restrictions as embodied sumud | Embodied: physical engagement with place and work; Economic: livelihood continuity as resilience; Cultural: occupational identity and tradition; Collective: community solidarity around shared risks | Highlights occupational identity as therapeutic resource; informs occupational mental health frameworks; demonstrates how livelihood and place attachment can anchor clinical formulation; relevant to humanitarian mental health in resource-restricted settings | Anthropology / Occupational Studies |
| 16. **Hamamra (2025)** | Gaza (youth cultural infrastructure; theatre and music) | Qualitative cultural analysis | Young people, educators, and cultural workers in Gaza | Theatre and music as survival pedagogy: sumud enacted through arts education as youth testimony, collective witnessing, and political identity formation; cultural erasure as counter-strategy requiring active artistic endurance | Cultural: arts as survival pedagogy; Collective: communal artistic production; Developmental: youth identity formation through creative expression; Political: cultural resistance to erasure | Strongly supports group arts-based therapies and expressive psychosocial interventions for youth; demonstrates how creative testimony can serve a witnessing and healing function; informs trauma-informed arts education | Cultural Studies / Education |
| 17. **Hamdonah & Joseph (2024)** | Palestinian diaspora communities (online; global) | Netnography (digital ethnography) | Palestinian youth and adults in diaspora (social media communities) | Dabke (Palestinian folk dance) in diaspora as sumud: digital and in-person performance of collective dance as re-embodied cultural continuity, place attachment, and resistance to erasure across displacement and distance | Embodied: dance as physical connection to cultural identity; Cultural: collective artistic practice as identity continuity; Digital: online community as diaspora resilience infrastructure; Relational: shared movement as bonding ritual | Supports embodied and movement-based therapies (dance/movement therapy); demonstrates group expressive work as diaspora mental health resource; highlights digital community as therapeutic support structure; informs culturally responsive group therapy | Media Studies / Cultural Studies |
| 18. **Hammad & Tribe (2021a)** | Gaza Strip (community and clinical settings) | Qualitative — interviews and focus groups | Palestinian adults in Gaza | Adaptive coping as sumud: endurance sustained through meaning-making, faith, social ties, and everyday acts of persistence; sumud operationalised as a collective coping orientation rather than an individual strategy | Psychological: meaning, faith, perceived control; Social: social support and solidarity; Cultural: religious practice as coping resource; Collective: shared framing of adversity as sustaining | Directly informs strength-based, culturally sensitive clinical practice; supports integration of religious and spiritual dimensions into MH assessment; relevant to narrative and existential therapy; cautions against decontextualised individualised CBT | Psychology / Counselling |
| 19. **Hammad & Tribe (2021b)** | Occupied Palestinian territories (narrative review scope) | Narrative literature review | — (review of existing literature; no primary population) | Sumud as culturally informed resilience: synthesises how sumud has been conceptualised across disciplines as collective, ethical, and culturally embedded form of endurance; critiques Western resilience models as insufficient for Palestinian context | Collective: community as primary resilience unit; Cultural: traditions, language, history as resources; Ethical: moral dimensions of endurance; Institutional: civil society and educational structures | Critiques and extends Western psychotherapy models; advocates for cultural humility in MH practice; supports development of culturally informed therapeutic frameworks; foundational review for sumud in clinical contexts | Psychology / Transcultural Psychiatry |
| 20. **Harrold (2020)** | Psychoanalytic and community psychology (theoretical) | Conceptual / psychoanalytic analysis | Communities under political violence (conceptual) | Sumud as ethical stance and moral witnessing: non-violent endurance as psychologically meaningful position; the analytic concept of 'holding on' as psychic equivalent of sumud; collective mourning and sumud as related processes | Psychological: mourning, ethical agency, holding on; Moral: non-violent endurance as meaningful stance; Collective: communal psychological processes; Relational: witnessing as therapeutic act | Applies psychoanalytic and existential frameworks to collective sumud; supports dignity-centred, psychodynamic, and group analytic approaches; affirms value of moral witnessing in clinical work; relevant to trauma therapy and advocacy-informed practice | Psychology / Psychoanalytic Studies |
| 21. **Isa (2023)** | Jerusalem (urban precarious living contexts) | Ethnographic essay / urban sociology | Palestinian residents of East Jerusalem | Sumud as presence amid insecurity: endurance of precarious residency, fear of displacement, and administrative pressures through assertion of ordinary life; sumud as existential claim to belonging in contested urban space | Existential: being-in-place as resilience; Psychological: fear, uncertainty, and coping with unresolved threat; Spatial: urban environment as site of endurance; Cultural: community ties as anchor against displacement | Highlights clinical complexity of chronic, unresolved existential threat; supports therapeutic approaches that validate fear without pathologising it; informs formulation of patients facing housing insecurity and displacement; relevant to existential psychotherapy | Political Sociology / Urban Studies |
| 22. **Khodary et al. (2020)** | Turkey, Iraq, and Palestinian territories (feminist qualitative scope) | Feminist qualitative analysis | Iraqi, Palestinian, and Kurdish women | Gendered sumud: women's endurance as located at intersection of political oppression and gender-based constraint; sumud both enables women's agency and imposes burden; critique of romanticising women's endurance without attending to structural violence | Gendered: intersection of gender and political adversity; Ethical: tensions between agency and imposed obligation; Psychological: cumulative burden and distress; Relational: women as carers and cultural transmitters | Advocates for gender-sensitive mental health care; cautions against idealising sumud in ways that silence women's distress; supports feminist therapeutic approaches; relevant to trauma-informed gender-responsive practice | Sociology / Feminist Studies |
| 23. **Kteily-Hawa et al. (2020)** | Gaza Strip (community sample) | Quantitative cross-sectional survey | Adult Palestinians in Gaza (large community sample, n = 1,000+) | Sumud measured through resilience scales in a social-ecological framework: resilience scores examined in relation to sociodemographic factors, community resources, and emotional responses; sumud implicit as collective resilience orientation | Ecological: community resources and social environment; Individual: resilience scores; Sociodemographic: education, gender, age as moderators; Emotional: affective responses to chronic adversity | Demonstrates empirical measurability of resilience in conflict context; highlights community resources (not individual traits) as strongest predictors; informs community-level mental health programming; supports social-ecological models over individual deficit approaches | Public Health / Epidemiology |
| 24. **Lax (2021)** | ASHTAR Theatre (Ramallah, West Bank) | Case study / theatre studies | Palestinian theatre artists and practitioners | Creative endurance as sumud: ASHTAR Theatre's sustained artistic production under occupation reframed as sumud — an active rejection of resilience as passive survival and an assertion of cultural vitality as ethical stance | Cultural: artistic production as ethical act; Collective: theatre community as sumud collective; Creative: expression as endurance; Moral: rejection of victimhood framing | Demonstrates value of arts-based therapy and creative expression groups; supports drama therapy and performance-based psychosocial work; highlights how creative communities can function as therapeutic and resilience-sustaining structures | Theatre Studies / Performance Studies |
| 25. **Marie et al. (2016)** | West Bank, occupied Palestinian territory | Narrative review / mental health systems analysis | Palestinian population (system-level review) | Social-ecological resilience as basis for MH system design: sumud implicit in the framing of community and cultural assets as foundations for service development; reviews MH needs and service gaps through a resilience lens | Institutional: MH service infrastructure; Ecological: community resources; Cultural: Palestinian identity as context for care; Collective: community as service and support unit | Directly informs mental health service planning in conflict settings; contextualises clinical care within broader social-ecological resilience framework; advocates for culturally responsive, community-based service design | Psychiatry / Mental Health Systems |
| 26. **Marie et al. (2017)** | Community mental health centres, West Bank | Qualitative — interviews and observations | Palestinian mental health nurses (community MH workforce) | Professional sumud: nurses sustain mental health practice under occupation through ethical commitment, collegial solidarity, shared professional identity, and moral purpose; professional identity as component of sumud | Professional: ethical commitment, occupational identity; Relational: collegial support and solidarity; Psychological: maintaining purpose under high demand; Moral: nursing as form of sumud | Directly relevant to staff wellbeing, supervision, and workforce resilience; supports reflective practice, peer supervision, and group-based staff support; affirms moral injury framework for health professionals in conflict; informs clinical leadership in humanitarian settings | Psychiatry / Nursing |
| 27. **Marie et al. (2018)** | Palestinian communities (West Bank; conceptual-empirical mixed) | Qualitative and theoretical analysis | Palestinian community members and professionals | Social ecology of sumud: conceptualises sumud as relationally and ecologically embedded resilience, integrating individual, family, community, and institutional levels; develops a social-ecological framework for understanding sumud as collective endurance | Ecological: multilevel resilience (individual → institutional); Relational: family and community as primary resilience units; Institutional: civil society and MH services; Cultural: sumud as shared ethical orientation across levels | Foundational for systemic psychotherapy and family-based approaches; provides an integrative ecological framework for clinical formulation; supports multilevel MH interventions; challenges individually focused treatment models | Psychiatry / Social Ecology |
| 28. **Mason & Dajani (2019)** | Occupied Golan Heights (Druze communities) | Conceptual ethnography / political ontology | Druze communities under Syrian and Israeli administration | Place-based sumud: land attachment and territorial identity as foundations of collective endurance; sumud as ontological (being-in-place) rather than merely political; warns against exporting Palestinian sumud framework uncritically to other contexts | Ontological: belonging and place as resilience; Cultural: land-based identity; Collective: community as territorial and cultural unit; Political: sumud adapted across different sociopolitical contexts | Cautions against individualising sumud or applying it decontextually; supports place-based and community-rooted therapeutic approaches; relevant to formulation with patients for whom land, home, and territory hold deep identity significance | Political Studies / Anthropology |
| 29. **Qudaih et al. (2025)** | Gaza Strip (hospital and clinical settings; 2023–24 conflict) | Quantitative survey | Physicians and nurses working in Gaza during active conflict | Professional endurance as sumud: health-care workers sustain practice under catastrophic conditions through professional identity, moral purpose, and collective responsibility; measures psychological strain alongside endurance | Professional: moral purpose, occupational identity; Psychological: psychological strain, compassion fatigue; Relational: colleague support; Collective: shared mission as protective | Critically important for staff support and clinical supervision frameworks; informs moral injury-informed supervision, peer support models, and burnout prevention in humanitarian health-care settings; relevant to workforce mental health policy | Public Health / Occupational Health |
| 30. **Roy (2016)** | Ramallah and Rawabi (West Bank urban settings) | Theoretical / postcolonial urban analysis | Palestinian urban residents and professionals | Everyday urban resilience as sumud: negotiation of identity, class, and modernity in contemporary Palestinian cities; critiques romanticised sumud narratives that obscure class dynamics and capitalist urbanism; calls for contextually nuanced resilience analysis | Urban: spatial and material dimensions of resilience; Class: socioeconomic differentiation within sumud; Cultural: modern Palestinian identity; Critical: deconstructs homogeneous sumud narratives | Cautions against uncritical clinical application of sumud; supports intersectional formulation that attends to class, urbanity, and social position; relevant to social-determinants-informed clinical practice and community mental health | Postcolonial Studies / Urban Geography |
| 31. **Ryan (2015)** | West Bank (community and domestic settings) | Feminist qualitative — interviews and ethnography | Palestinian women (community sample; mixed age and background) | Everyday resilience as resistance: women's daily acts — homemaking, caregiving, maintaining routines, sustaining family life — conceptualised as sumud; demonstrates that sumud is not extraordinary heroism but ordinary ethical endurance; warns against romanticising burden as strength | Gendered: women's everyday labour as resilience; Ethical: moral dimensions of caregiving; Relational: family as site of endurance; Critical: tensions between agency and imposed responsibility | Foundational for feminist-informed and gender-responsive psychotherapy; cautions against therapeutic approaches that valorise suffering; supports validation of distress alongside acknowledgement of strength; informs trauma-informed feminist practice | Political Sociology / Gender Studies |
| 32. **Sazzad (2016)** | Palestinian literary tradition (Mahmoud Darwish poetry) | Literary analysis / cultural studies | Palestinian literary and cultural communities (via Darwish's work) | Sumud in poetry as cultural resistance: Mahmoud Darwish's poetry analysed as embodiment of sumud — linguistic, symbolic, and aesthetic endurance that sustains collective Palestinian identity across displacement and loss | Symbolic: poetic language as resilience; Cultural: literary heritage as collective memory; Aesthetic: beauty as act of endurance; Intergenerational: poetry transmitted across generations | Supports narrative and meaning-centred therapy; demonstrates how poetry and literary heritage can anchor identity in therapeutic work; relevant to creative writing and bibliotherapy in MH contexts; informs culturally sensitive formulation for patients with strong literary/cultural identity | Cultural Studies / Literary Studies |
| 33. **Scott et al. (2025)** | Palestine and diaspora (digital learning communities) | Conceptual and practice-based analysis | Palestinian learners, educators, and digital community members | Sumud as connected learning: digital commons and networked learning communities as 21st-century expressions of sumud; online educational infrastructures sustain collective identity, knowledge, and pedagogical continuity across fragmentation and displacement | Digital: online community as resilience infrastructure; Educational: learning continuity as sumud; Collective: distributed community maintaining shared purpose; Cultural: digital practices as identity preservation | Demonstrates relevance of digital and online group approaches in mental health; supports digital group therapy and peer support platforms for displaced and diaspora communities; informs telemental health and e-learning-based psychosocial programmes | Education / Digital Learning Studies |
| 34. **Taha et al. (2025)** | Gaza (youth parkour communities) | Qualitative — interviews and field observations | Young male parkour practitioners in Gaza | Embodied decolonial sumud: parkour practice in Gaza as land-based physical activity through which young men enact attachment to place, reclaim bodily agency, and sustain connection to contested urban space; movement as sumud | Embodied: body-based assertion of place and agency; Physical: movement as resilience practice; Spatial: engagement with built environment as attachment; Developmental: youth identity through physical challenge | Supports body-oriented and movement-based therapies (somatic, sport-based interventions); demonstrates how physical activity can anchor identity and agency in MH work; relevant to sport-based psychosocial programmes for young men; informs occupational and activity-based therapy | Sociology / Sport Studies |
| 35. **Underhill (2022)** | Palestinian visual art (archival and contemporary practice) | Cultural analysis / visual art studies | Palestinian visual artists and archivists | Archival sumud: Palestinian visual artists sustain collective memory and cultural continuity through reference to, remixing of, and reverence for archival materials; art-making as a form of sumud that preserves and transmits identity across loss | Cultural: archival practice as identity maintenance; Artistic: visual art as memory and mourning; Intergenerational: transmission of cultural heritage; Collective: art community as resilience group | Supports arts-based therapies involving visual expression, collage, and archive work; demonstrates therapeutic potential of working with personal and family photographs, documents, and objects; informs trauma-informed arts therapy and narrative approaches using material culture | Visual Art / Cultural Studies |
| 36. **Veronese et al. (2021)** | Occupied Palestinian territory (West Bank; clinical and community settings) | Qualitative — individual and collective life events calendars | Palestinian women (mixed community and professional sample) | Sumud as meaning-based coherence: survival and resilience among Palestinian women sustained through narrative coherence, ethical identity, and professional purpose; life events calendar methodology reveals how sumud is lived chronologically and relationally | Psychological: narrative coherence, sense of purpose; Ethical: professional identity and moral responsibility; Relational: collective solidarity among women; Methodological: life events calendar as tool for resilience mapping | Directly supports existential and meaning-centred psychotherapy; life events calendar methodology is directly transferable to clinical assessment; affirms women-centred group therapy and narrative approaches; relevant to transcultural psychiatric practice | Transcultural Psychiatry / Psychology |
| 37. **Wick (2008)** | West Bank (maternity and reproductive health settings) | Ethnography — birth and reproductive care | Palestinian women and families (perinatal context) | Reproductive sumud: childbirth and reproductive care under occupation as site of sumud; maintaining reproductive health infrastructure as institutional endurance; birth as political and cultural affirmation of continuity and future | Embodied: reproductive body as site of endurance; Institutional: health infrastructure maintenance; Cultural: birth as affirmation of communal future; Gendered: women's reproductive labour as sumud | Informs perinatal mental health care in conflict settings; demonstrates importance of culturally embedded reproductive care; supports midwifery-led and community-based perinatal MH approaches; relevant to gender-sensitive and culturally responsive clinical care | Cultural Psychiatry / Medical Anthropology |
| 38. **Aqtam, Shouli & Ayed (2026)*** | Healthcare settings, West Bank (conflict setting) | Qualitative — in-depth interviews | Palestinian nurses working under occupation and systemic violence | Professional endurance as moral witnessing: nurses sustain clinical practice under conditions of dual jeopardy — as healthcare providers and as survivors of the same systemic violence affecting their patients; sumud enacted through maintenance of professional role, ethical commitment, and relational care despite personal loss and threat | Professional: occupational identity and moral purpose as anchors of endurance; Psychological: cumulative trauma, grief, and vicarious loss alongside continued function; Relational: collegial solidarity and shared professional burden; Ethical: obligation to patients framed as moral expression of sumud; Gendered: nursing workforce gendered dynamics under occupation | Critically important for staff support, reflective practice, and clinical supervision frameworks in conflict settings; directly informs moral injury-informed supervision and peer support models; cautions against romanticising professional endurance or normalising harm; supports workforce mental health policy in humanitarian healthcare; relevant to burnout prevention and compassion fatigue frameworks | Nursing / Transcultural Psychiatry |
| 39. **McMahon et al. (2026)*** | Gaza and occupied Palestinian territories (conceptual scope; international humanitarian context) | Conceptual / narrative review | Population-level (Palestinian civilians; conflict-affected communities; mental health workforce) | Sumud as culturally rooted collective protective factor against the individualising and depoliticising tendencies of Western mental health models; framed within liberation psychology and decolonial mental health; sumud positioned as a communal and ethical orientation that sustains dignity, solidarity, and collective endurance under structurally produced suffering; Palestinian distress explicitly reframed as a rational and politically intelligible response to ongoing violence rather than psychopathology | Collective: community solidarity and family cohesion as primary resilience structures; Cultural: sumud as an embedded orientation within Palestinian social and cultural life; Ethical: dignity, justice, and self-determination as dimensions of psychological health; Structural: structural violence and political conditions foregrounded as determinants of distress; Institutional: community-based and rights-oriented service frameworks advocated | Reframes Palestinian distress as a rational response to systemic violence rather than individual psychopathology; advocates for community-based, dignity-centred, and rights-informed mental health responses; critiques individualising psychiatric models as epistemically and ethically inadequate in conflict settings; strongly supports decolonial, liberation-psychology-informed, and culturally responsive clinical frameworks; relevant to formulation, therapeutic stance, and advocacy-informed clinical practice | Psychiatry / Global Mental Health / Transcultural Psychiatry |

*Notes: Study references correspond to the Vancouver-numbered reference list in the main manuscript. Column 5 (Conceptualisation of Sumud) records the primary definition or operationalisation of sumud as used in each study. Column 6 (Key Resilience Dimensions) records dimensions identified by primary extractors at the psychological, relational, cultural, embodied, or institutional level. Column 7 (MH / Psychotherapy Relevance) records both direct clinical implications and relevant cautions as reported or inferable from each study. N/A in any cell indicates the domain was not addressed in the primary source.*
